# MC1R Gene Variants and Their Relationship with Coat Color in South American Camelids

**DOI:** 10.1155/2023/4871135

**Published:** 2023-08-30

**Authors:** Carola Melo Rojas, P. Walter Bravo Matheus, Celso Zapata Coacalla, Victor Lopez Durand, Maximo Melo Anccasi

**Affiliations:** ^1^Laboratorio de Genetica, Escuela Profesional de Medicina Veterinaria Canchis, National University of Saint Anthony the Abbot in Cuzco, Cusco, Peru; ^2^Facultad de Medicina Veterinaria y Zootecnia, Universidad Nacional del Altiplano, Puno, Peru

## Abstract

In domestic camelids, fleece color is an essential characteristic because it defines the direction of production. Variants were determined in the *MC1R* gene that showed a relationship with coat color in alpacas and llamas at the level of the coding region. This report sequenced the *MC1R* gene from 290 alpacas (142 white, 84 black, 50 brown, and 14 light fawn), five brown llamas, nine vicuñas, and three guanacos to analyze the association between coat color and the *MC1R* gene among South American camelids. A total of nineteen polymorphisms were identified. Seven polymorphisms were significant; three of them were of nonsynonymous type (c.82A > G, c.376G > A, and c.901C > T), two were of synonymous type (c.126 T > C and c.933G > A), one was in the promoter region (−42C > G), and one was in the 3′ UTR (+5T > C). More polymorphisms were found in domestic camelids than in wild camelids. Besides polymorphism, the association of polymorphisms might cause white and dark pigmentation in the fleece of South American camelids. In addition, the MC1R protein would answer the pigmentation in alpacas.

## 1. Introduction

Mammals have distinct colors, which are determined by the biochemical function, distribution, and availability of pheomelanin and eumelanin pigments. Pheomelanin gives red and yellow color, while eumelanin gives black and brown phenotypes [1]. The discovery and analysis of candidate genes associated with coat color led to a better understanding of pigmentation mechanisms [2]. More than 150 genes associated with color have been identified with or without epistatic interactions [3]. In this sense, the extension locus encodes the melanocortin one receptor (*MC1R*) expressed in the hair follicle and skin melanocytes. Melanocyte-stimulating hormone activates the melanocytic activity of *MC1R*, and mutations at some single-nucleotide polymorphism (SNP) loci have been shown to alter mammalian coat color in guinea pigs [4] and the chestnut horse [5]. On the other hand, the mutation of the *MC1R* gene has been linked to different colors in pigs [6, 7], cattle [8, 9], sheep [10, 11], goats [12], dogs [13, 14], chicken [15, 16], and foxes [17, 18]. In those cases, a functional mutation increased to give a black/dark color, and lack of function usually generated a light coat color.

Some studies in alpacas identified mutations in the *MC1R* gene that determine light phenotypes, but without being conclusive [19–21], and in the case of dromedaries, it is indicated that a polymorphism (c.901C > T) is typical of light-coated animals [2, 22].

This study aimed at analyzing the association between dark coat color and the candidate gene *MC1R* among South American camelids.

## 2. Materials and Methods

### 2.1. Animal Material

Three hundred seven unrelated camelids were studied for *MC1R* gene sequencing; 290 were alpacas (142 white, 84 black, 50 brown, and 14 light fawn), five brown llamas, nine vicuñas, and three guanacos. Animals were from three different sites in the Cusco region, two of them with significant recognition for the quality of the animals (Oquemarca in Phinaya and Chaupi Wasi in Maranganí) and the La Raya Research Center, UNSAAC, as well as samples from the Puno region (Huaycho and Corani). All the sites are located more than 4000 meters above sea level. The samples were obtained by venipuncture from the jugular vein into EDTA vacutainer tubes. Blood was frozen at −20°C until handling at the La Raya Research Center from Universidad Nacional de San Antonio Abad del Cusco.

### 2.2. DNA Extraction

Deoxyribonucleic acid (DNA) was extracted from the whole blood of all sampled animals using the PureLink Genomic Mini Kit (Qiagen) according to the manufacturer's protocol.

### 2.3. Primer Design and Amplification

Polymerase chain reaction (PCR) primers to amplify the coding region and 5′ and 3′ regions were designed with the Ion AmpliSeq 6.0.1 designer for Ion Torrent new generation (Life Technologies, https://www.ampliseq.com) from the VicPac3.1 reference sequence. A total of 5 amplicons ranging from 275 bp to 335 bp covered 100% of the target sequence (Table 1). The primers were supplied in two pools, each at a concentration of 100 nM (Life Technologies, Carlsbad, CA, USA).

### 2.4. Sequencing of Candidate Genes

The sequencing library was prepared with the Ion AmpliSeq Library Kit (version 2.0) according to the manufacturer's protocols for affected individual CSA110.03. The amplified library was diluted to 10 pM, and 25 *μ*L was used for template preparation according to the Ion PGM Template OT2 200 Kit protocol using the Ion PGM Template OT2 200 kit. The clonally amplified library was enriched on Ion OneTouch ES. Sequencing was performed on an Ion Torrent bulk sequencing machine using the Ion PGM Sequencing 200 v2 kit and an Ion 540 chip. Using Torrent Suite (version 3.6.56201) variant call format (VCF) files were uploaded to Ion Reporter V4.0 (https://ionreporter.lifetechnologies.com/ir/) for variant annotation.

### 2.5. Variant Analysis

The first run of 168 samples was performed on Ion Torrent Variant Caller (TVC). Those variants were combined using Genome Analysis Tool Kit (GATK), and the resulting VCF file had 20 variants. Variant calling was then rerun with 344 samples using the list of variants generated in the first pass as hotspots for the variants. This procedure allowed us to detect variants and spots with the reference allele. The final matrix with all samples had 19 variants. The MC1R gene sequences were deposited in GenBank with accession numbers MT789859–MT790208. The sequences were aligned using the software Bosque 2.0.2 [23]. Two software programs, DNAsp 6.0 [24] and Network v.10.1.0.0. (Fluxus Technology Ltd.), were employed to construct a haplotype network [25].

### 2.6. Statistical Analysis

The Beagle 4.1 software was used to perform the imputation of nongenotyped tags; the resulting file was processed with PLINK v1.90b4.7 using MAF filters of 0.05, MIND 0.5, HWE 0.001, and GENO 0.2. Minor allele frequency criteria, Hardy–Weinberg equilibrium criteria, and missing genotype criteria filtered 19 variants. Finally, the PLINK association test for fleece color was used. Variants were analyzed with a *P* value adjusted by the Holm–Bonferroni method.

## 3. Results

### 3.1. Phenotypic Characterization

**Figure 1:**
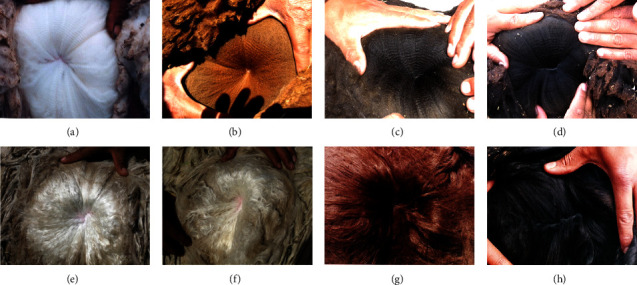
Fleeces of different colors in alpacas. (a) White fleece of Huacaya alpaca, (b) brown fleece of Huacaya alpaca, (c) bay black fleece of Huacaya alpaca, (d) black fleece of Huacaya alpaca, (e) white fleece of Suri alpaca, (f) light fawn fleece of Suri alpaca, (g) dark brown fleece of Suri alpaca, and (h) black fleece of Suri alpaca.

The phenotypic characteristics of alpacas with fleeces ranging from white to black, llamas with brown and white coats, and vicuñas and guanacos, some of the colors analyzed, are presented in Figures 1 and 1S.

### 3.2. Genetic Analysis

A total of 1238 bp corresponding to the alpaca MC1R gene, including the 5′ and 3′ UTR regions, were determined from 307 animals (Tables 1S and 2S). Seventeen SNP polymorphisms and two indels were also identified. Six were silent mutations, seven were nonsense polymorphisms, and one was in the 5′ region; there were also a four-base pair deletion, another one-base pair deletion, a five-base pair insertion, and two polymorphisms in the 3′ UTR (Table 2 and Figure 2S). Five of the nineteen polymorphisms were unreported and are presented in Table 2. The comparison between genotypes and phenotypes to determine the association with coat color is shown in Table 3.

Strong associations with coat color were found only in eight polymorphisms (Table 3) from genotyping data (Table 3S): three involved amino acid change (c. 82A > G, c. 376G > A, and c. 901C > T), two involved synonymous changes (c.126 T > C and c. 933G > A), one located in the 5′ region (5′c.−42C > G), and two in the 3′ UTR (c. +5T > C and +170 G> C; Tables 3 and 4S with more details of the analyses performed). Forty-one haplotypes were identified (Table 5S) with a haplotypic diversity of 0.9523. Likewise, *MC1R* gene sequence translation revealed an amino acid size of 318 amino acids.

Four polymorphisms detected in alpacas (c. 92C > T, c. 239-243ins, c. 243 C > T, and c. 629T) were not found in vicunas and guanacos. Two polymorphisms were present in alpacas and vicuñas, but not in llamas and guanacos (c. 72G > C and c. 265A > G). Six polymorphisms present in alpacas, llamas, and guanacos were absent in vicuñas (c. 82A > G, c.126 T > C, c. 383T > C, c. 933G > A, 5′c.−42C > G, and 3′UTR +5T > C), and three polymorphisms were present in alpacas and llamas but not in wild camelids (c. 224-227del, c. 259A > G, and c. 376G > A).

## 4. Discussion

The present study of the *MC1R* gene in South American camelids allowed a broader overview since a complete genome was used. In this sense, amplifying a large region of the MC1R gene was possible, providing, at the same time, a better understanding of the expression of this gene with the actual color of animals. Thus, the results of the present study are consistent with those of previous reports [19–21, 26, 28, 30].

Although eight significant polymorphisms are reported (Table 3), three are of nonsynonymous type (c.82A < G, c.376G > A, and c.901C > T that caused amino acid change p.T28A, p.G126S, and p.R301C, respectively), two are of synonymous type (c.126T > G and c.933G> A), one polymorphism is in the 5′ region (−42C > G), and two polymorphisms are in the 3′ region (+8T > C and +170G > C). The three polymorphisms that were of nonsynonymous type have been responsible for the difference in tones in alpaca fleece, interacting in combination with each other for different color phenotypes where the combination of genotypes G82/C126/G − 42(5′) and genotypes T901/A933/C + 5/C + 170 and C901T/G933A/T + 8C/G + 170C has a better association for white fleece color (Table 2). In the case of brown fleece alpacas, the majority were heterozygous and homozygous for the total set of polymorphisms, and the three genotypes marked in white alpacas were not significant. It is also significant that the A376 polymorphism was found in 89.58% of white animals. Thus, this polymorphism could be used as a marker for color. In some species, there are several alleles at MC1R [29]. The authors in [19, 30] indicated that a combination of mutations at the MC1R locus could cause eumelanin and pheomelanin synthesis in alpacas. This assessment was corroborated in the present study.

The c.82A > G SNP genotypes were significantly associated with the color phenotypes analyzed. Some residues in this domain are critical in the normal functioning of the protein [31]. The authors in [20, 21] mentioned that there are a more significant number of alpacas with the A82G genotype than in the present study; they found a significant population of white alpacas with the G82 genotype. This finding could be due to the population analyzed and a more significant number of animals studied.

Although synonymous, the c.126T > C polymorphism was shown to play a significant role in fleece pigmentation in statistical analysis. This SNP is likely linked to a promoter mutation [32] and may be a good predictor for pheomelanic [20] and eumelanic animals.

The c.376G > A polymorphism occurring at codon 126 was also shown to play a role in alpaca fleece color in statistical analysis, whereas it could serve as a marker for color. This codon is in the middle portion of the third transmembrane fragment [31], where it alters the structure of the protein and affects its ability to function [33]. Although this polymorphism was mentioned in [20, 21, 29], all found no association with this polymorphism; this could be again due to the number of animals analyzed and the origin of alpacas and llamas. In this study, nonrelated animals were used, which may provide a better understanding of this polymorphism.

The c.901C > T polymorphism was the one with the highest statistical value in the present study, where white alpacas were found with the TT and CT genotypes (58.78% and 36.73%, respectively) and black and brown alpacas were found with CC and CT genotypes, with the majority being the C901 genotype. The same situation was also determined in llamas, guanacos, and vicuñas analyzed where all the animals were of the CC genotype. These results differ from those found by the authors in [21, 34] who did not see white alpacas with the TT genotype and only found heterozygous white alpacas. The authors in [20, 29] mentioned genotypes similar to those reported in the present study. In the case of dromedaries, it has been reported that the polymorphism located at position 901 is responsible for white pigmentation in a dominant manner because heterozygosity of the T allele at c901C > T is sufficient for a white phenotype to occur [25]. The c.901C > T polymorphism occurs at the C-terminal end of the protein and might affect the structure of C-terminal MC1R [35, 36]. Evaluating the C901 genotype in the two wild camelids could indicate that it is wild type or ancestral and its role in the presentation of fleece shades in domestic camelids.

Another polymorphism found in the coding region and located at the C-terminal end, which was significant to the statistical analysis, was the c.933G > A synonymous polymorphism. This SNP was found in a highly significant domain concerning the structural integrity and function of the receptor [35, 37]. This polymorphism was found in Peruvian alpacas [16] and domestic and wild camelids [26]. However, they do not mention its significance for association studies with coat color.

Finally, it was identified that polymorphisms present at the level of the 5′ region (−42C > G) and in the 3′ UTR (+5T > C, +170G> C) were significantly associated with coat color in alpacas; This fact was revealing on this little region and affected gene expression changes. In addition, its effect should be analyzed systematically. Mutations affecting promoter regions and specific transcription factors have increased melanin synthesis [38]; 3′ untranslated regions (3′ UTRs) contain a critical class of noncoding variants that might impact posttranscriptional and translational processes [39]. Although one of these polymorphisms (3′+5T > C) was reported in [19, 34], it was not significant compared to the results of the present study.

When polymorphisms present in domestic and wild camelids were analyzed, domestic species presented a higher number of polymorphisms than the case of vicuñas and guanacos. These results are consistent with those of [26], which indicated that there is higher variability in domestic camelids apparently caused by the effects of the selection to which they were subjected.

In conclusion, from many animals (307), 19 polymorphisms were identified and presented in Peruvian alpacas, llamas, guanacos, and vicuñas. In addition, an in-depth study of this gene by studying 5′ and 3′ UTRs demonstrated their role in the coat of these camelids. Only 14 polymorphisms have been reported in this gene in alpacas [20, 26]. The present study adds four new polymorphisms that might also participate in expressing eumelanic and pheomelanic animals. Furthermore, the present study has identified a combination of polymorphisms that might play an essential role in fleece shade, as well as the case of heterozygosity and homozygosity, and not a single polymorphism as the cause of fleece color in alpacas. However, they act together to define fleece color.

## Figures and Tables

**Table 1:** Primers used to amplify the coding region of the *MC1R* gene and 5′ and 3′ regions.

Amplicon	Primer	Location
AMPL2094177	Fw: CCACAGCTCTTCCTGGAGATTC Rv: CTGCATCTTCAAGAATTTCAACCTCT	137308,137330 137602,137628

AMPL2094179	Fw: GCCAGCATGTGGACATATAGCA Rv: ACGTCATGGATGTGCTCATCTG	137815,137837 138075,138097

AMPL2094181	Fw: AGGCAGCAGATGAAGTAATACATGG Rv: CCAGGGAGAAGGTGAGTGTGA	138211,138236 138525,138546

AMPL2094180	Fw: GGTAGCGCAGTGCATAGAAGAT Rv: CTGCCATCACCAAGAACCGCAA	137993,138015 138246,138268

AMPL2094178	Fw: CCACGATGGAGTTGCAGATGAT Rv: CTCCTCTGTCTCGTCAGCTTTTT	137570,137592, 137862,137885

All the primers were using the reference (GCF_000164845.3). Vicugna_pacos 3.1. Primary assembly. AMPL: amplicon; Fw: forward strand; Rv: reverse strand.

**Table 2:** Mutations observed in alpacas, llamas, guanacos, and vicuñas.

Observed SNP	Amino acid change	Amino acid effect	Type of substitution	References
c.72G > C	Leu	N/A	Synonym	[26]
c.82A > G	Thr/Ala	Polar to nonpolar	Nonsynonym	[19, 26]
c.92C > T	Thr/Met	Polar to nonpolar	Nonsynonym	
c.126T > C	Asp	N/A	Synonym	
c.224-227del	Change in the reading frame	Disp	Nonsynonym	[19]
c.239-243ins	Change in the reading frame	Disp.	Nonsynonym	This study
c.243C > T	Ala	N/A	Synonym	This study
c.259A > G	Val/Met	Polar to nonpolar	Nonsynonym	[19, 26]
c.265A > G	Met/Val	Polar to nonpolar	Nonsynonym	[26]
c.354T > C	Asn	N/A	Synonym	[19, 26]
c.376G > A	Gly/Ser	Polar to polar	Non synonym	
c.383T > C	Met/Thr	Polar to nonpolar	Nonsynonym	[26]
c.618G > A	Leu	N/A	Synonym	[19, 26]
c.629Tdel	Change in the reading frame	Desp	Nonsynonym	This study
c.901C > T	Arg/Cys	Changed to polar	Nonsynonym	[19, 26, 27]
c.933G > A	Glu	N/A	Synonym	[19, 26]
5′c.−42C > G	N/A	N/A	N/A	This study
3′ + 5T > C	N/A	N/A	N/A	[26]
3′ + 170G > C	N/A	N/A	N/A	This study

SNP: single-nucleotide polymorphism; Disp.: displacement; del: deletion; ins: insertion. Five new polymorphisms contributed to the study of the *MC1R* gene.

**Table 3:** Polymorphisms identified in the *MC1R* gene.

SNP observed	Allele	Alpacas phenotype				*P* value
		White	Brown	Light fawn	Black	
c.82A > G	AA	10	18	0	43	*P* < 0.00000001
	AG	12	22	2	34	
	GG	92	5	7	5	

c.126T > C	TT	10	20	2	43	*P* < 0.00000001
	TC	12	23	0	35	
	CC	106	5	7	5	

c.376G > A	GG	6	14	0	34	*P* < 0.00000001
	GA	9	30	3	49	
	AA	129	11	11	5	

c.901C > T	CC	11	26	2	58	*P* < 0.00000001
	CT	54	19	6	26	
	TT	82	5	5	2	

c.933G > A	GG	10	17	2	44	*P* < 0.00000001
	GA	39	25	6	35	
	AA	92	5	5	4	

5′c. −42C > G	CC	10	19	0	43	*P* < 0.00000001
	CG	13	23	2	34	
	GG	101	5	7	5	

3′UTR +5T > C	TT	10	17	2	43	*P* < 0.00000001
	TC	39	24	6	36	
	CC	86	5	4	4	

3′UTR +170G > C	GG	9	19	2	44	*P* < 0.00000001
	GC	39	25	6	40	
	CC	8	5	4	3	

Polymorphisms were significant for white, black, and brown coat colors in alpacas. Some of them are found at the level of the 5′ and 3′ UTRs, indicating that these regions are also important for gene characterization studies and their role with coat color.

## Data Availability

The data used to support the findings of this study are available upon request from the corresponding author.

## Supplementary Materials

Supplementary Materials Figure S1: some coat color in alpacas and llamas. Figure S2: structural organization of the *MC1R* gene with identified polymorphisms. Table S1: information from the animals used in this study, with significant polymorphisms in statistical analysis for the trait analyzed. Table S2: information from animals used in this study, with polymorphisms found in the *MC1R* gene that were not significant in the statistical analysis. Table S3: distribution of genotypes for polymorphisms among different color phenotypes. Table S4: statistical analyses used in the population studied. Table S5: frequency of haplotypes in the population studied.
