# Supplementary material for: MC1R Gene Variants and Their Relationship with Coat Color in South American Camelids
Source: ScientificWorldJournal. 2023 Aug 30;2023:4871135. doi: 10.1155/2023/4871135 (PMC10541998; doi:10.1155/2023/4871135)
Supplement: Supplementary Materials — Figure S1: some coat color in alpacas and llamas. Figure S2: structural organization of the MC1R gene with identified polymorphisms. Table S1: information from the animals used in this study, with significant polymorphisms in statistical analysis for the trait analyzed. Table S2: information from animals used in this study, with polymorphisms found in the MC1R gene that were not significant in the statistical analysis. Table S3: distribution of genotypes for polymorphisms among different color phenotypes. Table S4: statistical analyses used in the population studied. Table S5: frequency of haplotypes in the population studied. [file 4871135.f1.zip › SNPs Table S2 (1).pdf]

Table S2: Composite information about the camelids used in this study (color, ID, place, polimorfisms whe the position was used from VicPac3.1 alpaca reference genome. The SNPs in this table turned out to be significant for the character analyzed.

Specie	Color	ID	Place	c.901	3'+170	c.126	c.933	3'+8	5'-42	c.82	c.376
Alpaca	White	301	Marangani	CC	CC	CC	AA	CC	GG	GG	AA
Alpaca	White	114	Marangani	TT	CC	CC	AA	CC	GG	GG	AA
Alpaca	White	124	Marangani	N	CC	N	AA	N	N	N	N
Alpaca	White	128	Marangani	CT	GC	TC	GA	TC	CG	AG	GA
Alpaca	White	143	Marangani	CT	GC	N	GA	TC	N	N	AA
Alpaca	White	146	Marangani	TT	CC	CC	AA	CC	GG	GG	AA
Alpaca	White	147	Marangani	TT	CC	CC	AA	CC	GG	GG	AA
Alpaca	White	148	Marangani	CT	GC	CC	GA	TC	GG	GG	AA
Alpaca	White	149	Marangani	TT	CC	CC	AA	N	N	N	AA
Alpaca	White	152	Marangani	TT	CC	CC	AA	N	N	N	AA
Alpaca	White	154	Marangani	TT	CC	CC	AA	CC	GG	GG	AA
Alpaca	White	156	Marangani	CT	GC	CC	GA	TC	GG	GG	AA
Alpaca	White	158	Marangani	CT	CC	CC	AA	CC	GG	GG	AA
Alpaca	White	184	Marangani	TT	CC	CC	AA	CC	GG	GG	AA
Alpaca	White	185	Marangani	CT	GC	N	GA	TC	N	N	N
Alpaca	White	186	Marangani	TT	CC	CC	AA	CC	GG	GG	AA
Alpaca	White	189	Marangani	TT	CC	CC	AA	CC	GG	GG	AA
Alpaca	White	191	Marangani	TT	CC	CC	AA	CC	GG	GG	AA
Alpaca	White	192	Marangani	CT	CC	CC	AA	CC	GG	GG	AA
Alpaca	White	201	Marangani	CT	GC	TC	GA	TC	CG	AG	GA
Alpaca	White	203	Marangani	CT	GG	TT	GG	TT	CC	AA	GG
Alpaca	White	211	Marangani	CT	GC	TC	GA	TC	CG	AG	N
Alpaca	White	270	Marangani	CT	GC	N	GA	TC	N	N	N
Alpaca	White	274	La Raya	CC	GG	TT	GG	TT	CC	AA	GG
Alpaca	White	275	La Raya	CT	N	N	N	TC	N	N	N
Alpaca	White	276	La Raya	CT	GC	N	GA	N	N	N	N
Alpaca	White	278	La Raya	CT	GC	N	GA	TC	N	N	N
Alpaca	White	282	La Raya	CC	GG	TT	GG	TT	CC	AA	GG
Alpaca	White	283	La Raya	CC	GG	TT	GG	TT	CC	AA	GA
Alpaca	White	286	La Raya	CT	GC	TC	GA	TC	CG	AG	N
Alpaca	White	291	La Raya	CT	GC	TC	GA	TC	CG	AG	N
Alpaca	White	295	La Raya	CT	GC	N	GA	TC	CG	N	N
Alpaca	White	342	La Raya	CT	GC	N	GA	TC	N	N	N
Alpaca	White	344	La Raya	CT	GC	N	GA	TC	N	N	N
Alpaca	White	137	La Raya	TT	CC	CC	AA	CC	GG	GG	AA
Alpaca	White	138	La Raya	CT	GC	CC	GA	TC	GG	GG	AA
Alpaca	White	139	La Raya	TT	CC	CC	AA	CC	GG	GG	AA
Alpaca	White	140	La Raya	CT	GC	CC	GA	TC	GG	GG	AA
Alpaca	White	141	La Raya	TT	CC	CC	AA	CC	GG	GG	AA
Alpaca	White	142	La Raya	TT	CC	CC	AA	CC	GG	GG	AA
Alpaca	White	143	La Raya	TT	CC	CC	AA	CC	GG	GG	AA
Alpaca	White	144	La Raya	TT	CC	CC	AA	CC	GG	GG	AA
Alpaca	White	145	La Raya	TT	CC	CC	AA	CC	GG	GG	AA
Alpaca	White	146	La Raya	TT	CC	CC	AA	CC	GG	GG	AA
Alpaca	White	148	Phinaya	CT	GC	CC	GA	TC	GG	GG	AA
Alpaca	White	149	Phinaya	TT	CC	CC	AA	CC	GG	GG	AA
Alpaca	White	151	Phinaya	CT	GC	TC	GA	TC	CG	AG	AA
Alpaca	White	158	Phinaya	CT	CC	CC	AA	CC	GG	GG	AA
Alpaca	White	159	Phinaya	CT	GC	CC	GA	TC	GG	GG	AA
Alpaca	White	176	Phinaya	CC	GG	TT	GG	TT	CC	AA	GG
Alpaca	White	178	Phinaya	CC	GG	TT	GG	TT	CC	AA	GA

N: No lectured
Homozygous
Heterozygous

Alpaca	White	180	Phinaya	CC	GG	TT	GG	TT	CC	AA	GA
Alpaca	White	182	Phinaya	TT	CC	CC	AA	CC	GG	GG	AA
Alpaca	White	184	Phinaya	TT	CC	CC	AA	CC	GG	GG	AA
Alpaca	White	185	Phinaya	CT	GC	N	GA	TC	N	N	N
Alpaca	White	193	Phinaya	TT	CC	CC	AA	CC	GG	GG	AA
Alpaca	White	194	Phinaya	CT	CC	CC	AA	CC	GG	GG	AA
Alpaca	White	106	Phinaya	TT	CC	CC	AA	CC	GG	GG	AA
Alpaca	White	109	Phinaya	TT	CC	CC	AA	CC	GG	GG	AA
Alpaca	White	113	Phinaya	TT	CC	CC	AA	CC	GG	GG	AA
Alpaca	White	117	Phinaya	CT	GC	TC	GA	TC	CG	AG	AA
Alpaca	White	120	Phinaya	TT	CC	CC	AA	CC	GG	GG	AA
Alpaca	White	122	Phinaya	TT	CC	CC	AA	CC	GG	GG	AA
Alpaca	White	124	Phinaya	TT	CC	N	AA	N	N	N	N
Alpaca	White	126	Phinaya	CT	GC	CC	GA	TC	GG	GG	AA
Alpaca	White	132	Phinaya	TT	CC	CC	AA	CC	GG	GG	AA
Alpaca	White	136	Phinaya	CT	GC	TC	GA	TC	CG	AG	AA
Alpaca	White	139	Phinaya	CT	CC	CC	AA	CC	GG	GG	AA
Alpaca	White	140	Phinaya	CT	GC	CC	GA	TC	GG	GG	AA
Alpaca	White	143	Phinaya	TT	CC	CC	AA	CC	GG	GG	AA
Alpaca	White	146	Phinaya	TT	CC	CC	AA	CC	GG	GG	AA
Alpaca	White	147	Phinaya	TT	CC	CC	AA	CC	GG	GG	AA
Alpaca	White	149	Phinaya	CT	GC	CC	GA	TC	GG	GG	AA
Alpaca	White	152	Phinaya	CT	GC	TC	GA	TC	CG	AG	AA
Alpaca	White	153	Phinaya	TT	CC	CC	AA	CC	GG	GG	AA
Alpaca	White	154	Nuñoa	TT	CC	CC	AA	CC	GG	GG	AA
Alpaca	White	155	Nuñoa	TT	CC	CC	AA	CC	GG	GG	AA
Alpaca	White	156	Nuñoa	CT	GC	CC	GA	TC	GG	GG	AA
Alpaca	White	157	Nuñoa	TT	CC	CC	AA	CC	GG	GG	AA
Alpaca	White	158	Nuñoa	TT	CC	CC	AA	CC	GG	GG	AA
Alpaca	White	159	Nuñoa	TT	CC	CC	AA	CC	GG	GG	AA
Alpaca	White	160	Nuñoa	TT	CC	CC	AA	CC	GG	GG	AA
Alpaca	White	161	Nuñoa	TT	CC	CC	AA	CC	GG	GG	AA
Alpaca	White	162	Nuñoa	TT	GC	CC	GA	TC	CG	AG	GA
Alpaca	White	164	Nuñoa	TT	CC	CC	AA	CC	GG	GG	AA
Alpaca	White	134	Nuñoa	CT	CC	CC	AA	CC	GG	GG	AA
Alpaca	White	137	Nuñoa	TT	CC	CC	AA	CC	GG	GG	AA
Alpaca	White	138	Nuñoa	TT	CC	CC	AA	CC	GG	GG	AA
Alpaca	White	121	Nuñoa	CC	GG	TT	GG	TT	CC	AA	GG
Alpaca	White	122	Nuñoa	TT	CC	CC	AA	CC	GG	GG	AA
Alpaca	White	123	Nuñoa	TT	CC	CC	AA	CC	GG	GG	AA
Alpaca	White	126	Nuñoa	TT	CC	CC	AA	CC	GG	GG	AA
Alpaca	White	128	Nuñoa	CT	GC	TC	GA	TC	CG	AG	GA
Alpaca	White	132	Nuñoa	TT	CC	CC	AA	CC	GG	GG	AA
Alpaca	White	146	Nuñoa	TT	CC	CC	AA	CC	GG	GG	AA
Alpaca	White	147	Nuñoa	CC	GG	TT	GG	TT	CC	AA	AA
Alpaca	White	148	Nuñoa	CT	GC	TC	GA	TC	CG	AG	GA
Alpaca	White	152	Nuñoa	CC	GG	TT	GG	TT	CC	AA	GG
Alpaca	White	154	Nuñoa	TT	CC	CC	AA	CC	GG	GG	AA
Alpaca	White	156	Nuñoa	CT	GC	CC	GA	TC	GG	GG	AA
Alpaca	White	109	Nuñoa	TT	CC	CC	AA	CC	GG	GG	AA
Alpaca	White	112	Nuñoa	TT	CC	CC	AA	CC	GG	GG	AA
Alpaca	White	113	Nuñoa	TT	CC	CC	AA	CC	GG	GG	AA
Alpaca	White	114	Nuñoa	TT	CC	CC	AA	CC	GG	GG	AA
Alpaca	White	115	Nuñoa	TT	CC	CC	AA	CC	GG	GG	AA
Alpaca	White	120	Nuñoa	CT	CC	CC	AA	CC	GG	GG	AA

Alpaca	White	158	Nuñoa	TT	CC	CC	AA	CC	GG	GG	AA
Alpaca	White	167	Nuñoa	TT	CC	CC	AA	CC	GG	GG	AA
Alpaca	White	168	Nuñoa	TT	CC	CC	AA	CC	GG	GG	N
Alpaca	White	177	Nuñoa	TT	CC	CC	AA	CC	GG	GG	AA
Alpaca	White	127	Nuñoa	TT	CC	CC	AA	CC	GG	GG	AA
Alpaca	White	129	Nuñoa	CT	GC	CC	GA	TC	GG	GG	AA
Alpaca	White	130	Nuñoa	CT	GC	CC	GA	TC	GG	GG	AA
Alpaca	White	131	Nuñoa	TT	CC	CC	AA	CC	GG	GG	AA
Alpaca	White	133	Nuñoa	CT	GC	CC	GA	TC	GG	GG	AA
Alpaca	White	134	Nuñoa	TT	CC	CC	AA	CC	GG	GG	AA
Alpaca	White	184	Nuñoa	TT	CC	CC	AA	CC	GG	GG	AA
Alpaca	White	186	Nuñoa	TT	CC	CC	AA	CC	GG	GG	AA
Alpaca	White	187	Nuñoa	TT	CC	CC	AA	CC	GG	GG	AA
Alpaca	White	189	Nuñoa	TT	CC	CC	AA	CC	GG	GG	AA
Alpaca	White	191	Nuñoa	TT	CC	CC	AA	CC	GG	GG	AA
Alpaca	White	192	Nuñoa	CT	CC	CC	AA	CC	GG	GG	AA
Alpaca	White	143	Macusani	CT	GC	N	GA	TC	N	N	AA
Alpaca	White	146	Macusani	TT	CC	CC	AA	CC	GG	GG	AA
Alpaca	White	147	Macusani	TT	CC	CC	AA	CC	GG	GG	AA
Alpaca	White	148	Macusani	CT	GC	CC	GA	TC	GG	GG	AA
Alpaca	White	149	Macusani	TT	CC	CC	AA	N	N	N	AA
Alpaca	White	152	Macusani	TT	CC	CC	AA	N	N	N	AA
Alpaca	White	157	Macusani	TT	CC	CC	AA	CC	N	N	AA
Alpaca	White	159	Macusani	CT	GC	N	GA	TC	N	N	GA
Alpaca	White	160	Macusani	CT	GC	TC	N	N	CG	N	GA
Alpaca	White	222	Macusani	TT	CC	CC	AA	CC	GG	GG	AA
Alpaca	White	223	Macusani	TT	CC	CC	AA	CC	GG	GG	AA
Alpaca	White	224	Macusani	TT	CC	CC	AA	CC	GG	GG	AA
Alpaca	White	225	Macusani	TT	CC	CC	AA	CC	GG	GG	AA
Alpaca	White	226	Macusani	TT	CC	CC	AA	CC	GG	GG	AA
Alpaca	White	227	Macusani	TT	CC	CC	AA	CC	GG	GG	AA
Alpaca	White	228	Macusani	TT	CC	CC	AA	CC	GG	GG	AA
Alpaca	White	229	Macusani	TT	CC	CC	AA	CC	GG	GG	AA
Alpaca	White	230	Macusani	CT	GC	CC	GA	TC	GG	GG	AA
Alpaca	White	231	Macusani	TT	CC	CC	AA	CC	GG	GG	AA
Alpaca	White	232	Macusani	TT	CC	CC	AA	CC	GG	GG	AA
Alpaca	Brown	154	Marangani	CT	GC	TC	GA	TC	CG	AG	GA
Alpaca	Brown	155	Marangani	CT	GC	TC	GA	TC	CG	AG	GA
Alpaca	Brown	156	Marangani	CT	GC	TC	GA	TC	CG	AG	GA
Alpaca	Brown	157	Marangani	CT	GC	TC	GA	TC	CG	AG	AA
Alpaca	Brown	174	Marangani	TT	CC	CC	AA	CC	GG	GG	AA
Alpaca	Brown	175	Marangani	TT	CC	CC	AA	CC	GG	GG	AA
Alpaca	Brown	102	Marangani	CC	GG	TT	GG	TT	CC	AA	GG
Alpaca	Brown	105	La Raya	CC	GG	TT	GG	TT	CC	AA	GG
Alpaca	Brown	111	La Raya	CT	GC	TC	GA	TC	CG	AG	GA
Alpaca	Brown	114	La Raya	CT	GC	TC	GA	TC	CG	AG	GA
Alpaca	Brown	118	La Raya	CT	GC	TC	GA	TC	CG	AG	GA
Alpaca	Brown	121	La Raya	CT	GC	TC	GA	TC	CG	AG	GA
Alpaca	Brown	123	La Raya	CC	GG	TT	GG	TT	CC	AA	GG
Alpaca	Brown	125	La Raya	CT	GC	TC	GA	TC	CG	AG	GA
Alpaca	Brown	127	La Raya	CT	GC	TC	GA	TC	CG	AG	AA
Alpaca	Brown	128	La Raya	CC	GG	TT	GG	TT	CC	AA	GG
Alpaca	Brown	129	La Raya	CC	GG	TT	GG	TT	CC	AA	GA
Alpaca	Brown	130	Phinaya	CT	GC	TC	GA	TC	CG	AG	GA
Alpaca	Brown	133	Phinaya	CT	GC	TC	GA	TC	CG	AG	GA

Alpaca	Brown	137	Phinaya	CT	GC	TC	GA	TC	CG	AG	GA
Alpaca	Brown	145	Phinaya	CT	GC	TC	GA	TC	CG	AG	GA
Alpaca	Brown	150	Phinaya	CT	GC	TC	GA	TC	CG	AG	GA
Alpaca	Brown	151	Phinaya	CC	GG	TT	GG	TT	CC	AA	GG
Alpaca	Brown	163	Phinaya	CT	GC	TC	GA	TC	CG	AG	GA
Alpaca	Brown	143	Macusani	CC	GG	TT	GG	TT	CC	AA	GG
Alpaca	Brown	124	Macusani	TT	CC	CC	AA	CC	GG	GG	AA
Alpaca	Brown	125	Macusani	TT	CC	CC	AA	CC	GG	GG	AA
Alpaca	Brown	153	Nuñoa	CC	GC	TC	GA	TC	CG	AG	GA
Alpaca	Brown	110	Nuñoa	CC	GG	TT	GG	TT	CC	AA	GG
Alpaca	Brown	132	Nuñoa	CC	GG	TT	GG	TT	CC	AA	GG
Alpaca	Brown	135	Nuñoa	CC	GG	TT	GG	TT	CC	AA	AA
Alpaca	Brown	185	Nuñoa	TT	CC	CC	AA	CC	GG	GG	AA
Alpaca	Brown	188	Nuñoa	CT	GC	TC	GA	TC	CG	AG	GA
Alpaca	Brown	150	Nuñoa	CC	GC	TC	GA	N	CG	N	GA
Alpaca	Brown	154	Nuñoa	CC	GG	TT	GA	TC	N	N	GA
Alpaca	Brown	155	Nuñoa	CC	GG	TT	N	N	CC	N	GA
Alpaca	Brown	156	Nuñoa	CC	GG	N	N	N	N	N	GG
Alpaca	Brown	201	Nuñoa	CT	GC	TC	GA	TC	CG	AG	GA
Alpaca	Brown	203	Nuñoa	CC	GG	TT	GG	TT	CC	AA	GA
Alpaca	Brown	204	Nuñoa	CC	GG	TT	GG	TT	CC	AA	GG
Alpaca	Brown	205	Nuñoa	CC	GC	TC	GA	TC	CG	AG	AA
Alpaca	Brown	206	Nuñoa	CC	GG	TT	GG	TT	CC	AA	GG
Alpaca	Brown	210	Nuñoa	CC	GC	TC	GA	TC	CG	AG	GA
Alpaca	Brown	211	Nuñoa	CC	GG	TT	GG	TT	CC	AA	GA
Alpaca	Brown	215	Nuñoa	CC	GC	TT	GA	TC	CC	AA	GA
Alpaca	Brown	218	Nuñoa	CC	GC	N	GA	TC	GG	GG	AA
Alpaca	Brown	219	Nuñoa	CC	GG	TT	GG	TT	CC	AA	GA
Alpaca	Brown	239	Nuñoa	CC	GG	TT	GG	TT	CC	AA	GG
Alpaca	Brown	244	Nuñoa	CC	GG	TT	GG	TT	CC	AA	GG
Alpaca	Brown	241	Nuñoa	CT	GC	TC	GA	TC	CG	AG	GA
Alpaca	LF	152	Marangani	CC	GG	N	GA	TT	N	N	GA
Alpaca	LF	160	Phinaya	CT	GC	N	GA	TC	N	N	N
Alpaca	LF	173	Phinaya	CT	GC	CC	GA	TC	CG	GG	GA
Alpaca	LF	179	Phinaya	CT	GC	TC	GA	TC	CG	AG	GA
Alpaca	LF	190	Nuñoa	TT	CC	N	AA	TC	N	N	GA
Alpaca	LF	112	Nuñoa	N	N	N	N	N	N	N	GA
Alpaca	LF	131	Nuñoa	TT	CC	CC	AA	TC	CG	GG	GA
Alpaca	LF	135	Nuñoa	CT	GC	TC	GA	TC	CG	AG	GA
Alpaca	LF	138	Nuñoa	TT	CC	CC	AA	TC	CG	GG	GA
Alpaca	LF	127	La Raya	TT	CC	CC	AA	TC	CG	GG	GA
Alpaca	LF	133	La Raya	TT	CC	CC	AA	TC	CG	GG	GA
Alpaca	LF	150	La Raya	CT	GC	CC	GA	TC	CG	GG	GA
Alpaca	LF	136	La Raya	CT	GC	CC	GA	TC	CG	GG	GA
Alpaca	LF	208	La Raya	CC	GG	N	GG	TT	N	N	GA
Alpaca	Black	183	Marangani	TT	CC	CC	AA	CC	GG	GG	AA
Alpaca	Black	195	Marangani	CC	GC	TC	GA	TC	CG	AG	GA
Alpaca	Black	196	Marangani	CT	CC	CC	AA	CC	GG	GG	AA
Alpaca	Black	134	Marangani	CC	CC	CC	AA	CC	GG	GG	AA
Alpaca	Black	141	Marangani	TT	CC	CC	AA	CC	GG	GG	AA
Alpaca	Black	142	Marangani	CC	GG	TT	GG	TT	CC	AA	GG
Alpaca	Black	148	Marangani	CC	GG	TT	GG	TT	CC	AA	GA
Alpaca	Black	133	Marangani	CC	GG	TT	GG	TT	CC	AA	GG
Alpaca	Black	151	Marangani	CT	GC	CC	GA	TC	GG	GG	AA
Alpaca	Black	111	Marangani	CT	GC	TC	GA	TC	CG	AG	GA

Alpaca	Black	125	Marangani	CT	GC	TC	GA	TC	CG	AG	GA
Alpaca	Black	126	Marangani	CT	GC	TC	GA	TC	CG	AG	GA
Alpaca	Black	190	Marangani	CT	GC	TC	GA	TC	CG	AG	GA
Alpaca	Black	145	Marangani	CC	GC	TC	GA	TC	CG	AG	GA
Alpaca	Black	151	Phinaya	CT	GC	TC	N	N	N	N	GA
Alpaca	Black	242	Phinaya	CT	GC	TC	GA	TC	CG	AG	GA
Alpaca	Black	243	Phinaya	CC	GG	TT	GG	TT	CC	AA	GG
Alpaca	Black	302	Phinaya	CT	GC	N	GA	TC	N	N	N
Alpaca	Black	303	Phinaya	CC	GG	TT	GG	TT	CC	AA	GG
Alpaca	Black	304	Phinaya	CT	GC	TC	GG	TC	CG	AG	GA
Alpaca	Black	305	Phinaya	CC	GG	TT	GG	TT	CC	AA	GG
Alpaca	Black	306	Phinaya	CC	GG	TT	GG	TT	CC	AA	GG
Alpaca	Black	307	Phinaya	CC	GC	TC	GA	TC	CG	AG	GA
Alpaca	Black	308	Phinaya	CC	GG	TT	GG	TT	CC	AA	GA
Alpaca	Black	309	Phinaya	CC	GG	TT	GG	TT	CC	AA	GG
Alpaca	Black	310	Phinaya	CC	GG	TT	GG	TT	CC	AA	GA
Alpaca	Black	312	La Raya	CT	GC	TC	GA	TC	CG	AG	GA
Alpaca	Black	313	La Raya	CT	GC	TC	GA	TC	CG	AG	GA
Alpaca	Black	314	La Raya	CT	GC	TC	GA	TC	CG	AG	GA
Alpaca	Black	315	La Raya	CT	GC	TC	GA	TC	CG	AG	GA
Alpaca	Black	316	La Raya	CC	GG	TT	GG	TT	CC	AA	GG
Alpaca	Black	317	La Raya	CC	GG	TT	GG	TT	CC	AA	GG
Alpaca	Black	318	La Raya	CT	GC	TC	GA	TC	CG	AG	GA
Alpaca	Black	320	La Raya	CC	GG	TT	GG	TT	CC	AA	GG
Alpaca	Black	321	La Raya	CC	GG	TT	GG	TT	CC	AA	GG
Alpaca	Black	341	La Raya	CC	GG	TT	GG	TT	CC	AA	GG
Alpaca	Black	342	La Raya	CC	GC	TC	GA	TC	CG	AG	GA
Alpaca	Black	343	La Raya	CC	GG	TT	GG	TT	CC	AA	GG
Alpaca	Black	344	La Raya	CC	GG	TT	GG	TT	CC	AA	GG
Alpaca	Black	345	La Raya	CC	GC	TC	GA	TC	CG	AG	GA
Alpaca	Black	346	La Raya	CC	GC	TC	GA	TC	CG	AG	GA
Alpaca	Black	347	La Raya	CT	GC	TC	GA	TC	CG	AG	GA
Alpaca	Black	348	La Raya	CT	GC	TC	GA	TC	CG	AG	GA
Alpaca	Black	349	La Raya	CC	GG	TT	GG	TT	CC	AA	GG
Alpaca	Black	352	La Raya	CT	GC	TC	GA	TC	CG	AG	GA
Alpaca	Black	353	Nuñoa	CT	GC	TC	GA	TC	CG	AG	GA
Alpaca	Black	355	Nuñoa	CC	GG	TT	GG	TT	CC	AA	GG
Alpaca	Black	356	Nuñoa	CC	GC	TC	GA	TC	CG	AG	GA
Alpaca	Black	357	Nuñoa	CC	GC	TC	GA	TC	CG	AG	GA
Alpaca	Black	358	Nuñoa	CC	GG	TT	GG	TT	CC	AA	GG
Alpaca	Black	359	Nuñoa	CT	GC	TC	GA	TC	CG	AG	GA
Alpaca	Black	360	Nuñoa	CC	GC	TC	GA	TC	CG	AG	GA
Alpaca	Black	361	Nuñoa	CC	GG	TT	GG	TT	CC	AA	GG
Alpaca	Black	362	Nuñoa	CT	GC	TC	GA	TC	CG	AG	GA
Alpaca	Black	363	Nuñoa	CC	GG	TT	GG	TT	CC	AA	GG
Alpaca	Black	364	Nuñoa	CC	GG	TT	GG	TT	CC	AA	GG
Alpaca	Black	365	Nuñoa	CC	GG	TT	GG	TT	CC	AA	GG
Alpaca	Black	366	Nuñoa	CC	GG	TT	GG	TT	CC	AA	GG
Alpaca	Black	367	Nuñoa	CC	GG	TT	GG	TT	CC	AA	GG
Alpaca	Black	368	Nuñoa	CT	GC	TC	GA	TC	CG	AG	GA
Alpaca	Black	369	Nuñoa	CT	GC	TC	GA	TC	CG	AG	GA
Alpaca	Black	370	Nuñoa	CC	GG	TT	GG	TT	CC	AA	GA
Alpaca	Black	371	Nuñoa	CT	GC	TC	GA	TC	CG	AG	GA
Alpaca	Black	372	Nuñoa	CC	GG	TT	GG	TT	CC	AA	GG
Alpaca	Black	373	Nuñoa	CC	GG	TT	GG	TT	CC	AA	GA

Alpaca	Black	374	Nuñoa	CC	GG	TT	GG	TT	CC	AA	GG
Alpaca	Black	375	Nuñoa	CT	GC	TC	GA	TC	CG	AG	GA
Alpaca	Black	376	Nuñoa	CT	GC	TC	GA	TC	CG	AG	GA
Alpaca	Black	377	Nuñoa	CC	GG	TT	GG	TT	CC	AA	GA
Alpaca	Black	378	Nuñoa	CC	GC	TC	GA	TC	CG	AG	GA
Alpaca	Black	379	Nuñoa	CC	GG	TT	GG	TT	CC	AA	GG
Alpaca	Black	380	Nuñoa	CC	GG	TT	GG	TT	CC	AA	GG
Alpaca	Black	381	Nuñoa	CC	GG	TT	GG	TT	CC	AA	GA
Alpaca	Black	382	Nuñoa	CC	GG	TT	GG	TT	CC	AA	GA
Alpaca	Black	383	Nuñoa	CC	GG	TT	GG	TT	CC	AA	GG
Alpaca	Black	384	Nuñoa	CC	GG	TT	GG	TT	CC	AA	GG
Alpaca	Black	385	Nuñoa	CC	GG	TT	GG	TT	CC	AA	GA
Alpaca	Black	386	Nuñoa	CC	GG	TT	GG	TT	CC	AA	GG
Alpaca	Black	387	Nuñoa	CC	GC	TC	GA	TC	CG	AG	GA
Alpaca	Black	388	Nuñoa	CC	GG	TT	GG	TT	CC	AA	GG
Alpaca	Black	389	Nuñoa	CC	GG	TT	GG	TT	CC	AA	GA
Alpaca	Black	390	Nuñoa	CC	GG	TT	GG	TT	CC	AA	GA
Alpaca	Black	391	Nuñoa	CC	GC	TC	GA	TC	CG	AG	GA
Alpaca	Black	392	Nuñoa	CC	GG	TT	GG	TT	CC	AA	GG
Guanaco	Brown	1	Nuñoa	CC	GG	TT	GG	TT	CC	AA	N
Guanaco	Brown	2	Nuñoa	CC	GG	TT	GG	TT	CC	AA	N
Guanaco	Brown	3	Nuñoa	CC	GG	TT	GG	TT	CC	AA	N
Llama	Brown	1	Nuñoa	CC	GG	TT	GG	TT	CC	AA	N
Llama	Brown	2	Nuñoa	CC	GG	TT	GG	TT	CC	AA	GA
Llama	Brown	3	Nuñoa	CC	GG	TT	GG	TT	CC	AA	N
Llama	Brown	4	Nuñoa	CC	GG	TT	GG	TT	CC	AA	GA
Llama	Brown	5	Nuñoa	CC	GG	TT	GG	TT	CC	AA	N
Vicuña	Brown	1	Marangani	CC	N	N	N	N	N	N	N
Vicuña	Brown	2	Marangani	CC	N	N	N	N	N	N	N
Vicuña	Brown	3	Marangani	CC	N	N	N	N	N	N	N
Vicuña	Brown	4	Marangani	CC	N	N	N	N	N	N	N
Vicuña	Brown	5	Marangani	CC	N	N	N	N	N	N	N
Vicuña	Brown	6	Marangani	CC	N	N	N	N	N	N	N
Vicuña	Brown	7	Marangani	CC	N	N	N	N	N	N	N
Vicuña	Brown	8	Marangani	CC	N	N	N	N	N	N	N
Vicuña	Brown	9	Marangani	CC	N	N	N	N	N	N	N
