# Supplementary material for: MC1R Gene Variants and Their Relationship with Coat Color in South American Camelids
Source: ScientificWorldJournal. 2023 Aug 30;2023:4871135. doi: 10.1155/2023/4871135 (PMC10541998; doi:10.1155/2023/4871135)
Supplement: Supplementary Materials — Figure S1: some coat color in alpacas and llamas. Figure S2: structural organization of the MC1R gene with identified polymorphisms. Table S1: information from the animals used in this study, with significant polymorphisms in statistical analysis for the trait analyzed. Table S2: information from animals used in this study, with polymorphisms found in the MC1R gene that were not significant in the statistical analysis. Table S3: distribution of genotypes for polymorphisms among different color phenotypes. Table S4: statistical analyses used in the population studied. Table S5: frequency of haplotypes in the population studied. [file 4871135.f1.zip › SNPs Table S3.pdf]

Table S3: Composite information about the camelids used in this study (color, ID, place, polimorfisms whe the position was used from VicPac3.1 alpaca reference genome. The SNPs in this table turned out to be not significant for the character analyzed.

Specie	Color	ID	Place	Code	Allele	Code	Allele	Code	Allele	Code	Allele	Code	Allele	Code	Allele	Code	Allele	Code	Allele	Code	Allele	Code	Allele
Alpaca	White	107	Marangani	c.629del	N	c.618	GA	c.383	TC	c.354	TC	c.265	N	c.259	GG	c239-243ins	N	c.224-227del	Absent	c.92	N	c.72	N
Alpaca	White	114	Marangani	c.629del	Absent	c.618	AA	c.383	CC	c.354	CC	c.265	GG	c.259	GG	c239-243ins	Absent	c.224-227del	Absent	c.92	TT	c.72	CC
Alpaca	White	127	Marangani	c.629del	N	c.618	N	c.383	CC	c.354	N	c.265	N	c.259	N	c239-243ins	Heterozygous	c.224-227del	N	c.92	TT	c.72	CC
Alpaca	White	128	Marangani	c.629del	N	c.618	N	c.383	CC	c.354	N	c.265	N	c.259	N	c239-243ins	N	c.224-227del	Absent	c.92	TT	c.72	CC
Alpaca	White	137	Marangani	c.629del	Absent	c.618	N	c.383	CC	c.354	CC	c.265	GG	c.259	GG	c239-243ins	Absent	c.224-227del	Absent	c.92	N	c.72	N
Alpaca	White	139	Marangani	c.629del	Absent	c.618	AA	c.383	CC	c.354	CC	c.265	GG	c.259	GG	c239-243ins	Absent	c.224-227del	Absent	c.92	N	c.72	N
Alpaca	White	142	Marangani	c.629del	Absent	c.618	GA	c.383	CC	c.354	TC	c.265	GG	c.259	GG	c239-243ins	Absent	c.224-227del	Heterozygous	c.92	N	c.72	N
Alpaca	White	143	Marangani	c.629del	Absent	c.618	GA	c.383	CC	c.354	TC	c.265	GG	c.259	GG	c239-243ins	Absent	c.224-227del	Heterozygous	c.92	N	c.72	N
Alpaca	White	146	Marangani	c.629del	N	c.618	N	c.383	CC	c.354	N	c.265	N	c.259	N	c239-243ins	Heterozygous	c.224-227del	N	c.92	TT	c.72	CC
Alpaca	White	147	Marangani	c.629del	N	c.618	N	c.383	CC	c.354	N	c.265	N	c.259	N	c239-243ins	Homozygous	c.224-227del	Absent	c.92	TT	c.72	CC
Alpaca	White	148	Marangani	c.629del	N	c.618	N	c.383	CC	c.354	N	c.265	N	c.259	N	c239-243ins	N	c.224-227del	N	c.92	TT	c.72	CC
Alpaca	White	149	Marangani	c.629del	Absent	c.618	AA	c.383	CC	c.354	N	c.265	GG	c.259	GG	c239-243ins	Absent	c.224-227del	Absent	c.92	TT	c.72	N
Alpaca	White	150	Marangani	c.629del	Absent	c.618	AA	c.383	CC	c.354	CC	c.265	GG	c.259	GG	c239-243ins	Absent	c.224-227del	Absent	c.92	N	c.72	N
Alpaca	White	152	Marangani	c.629del	Absent	c.618	AA	c.383	CC	c.354	N	c.265	GG	c.259	GG	c239-243ins	Absent	c.224-227del	Absent	c.92	TT	c.72	N
Alpaca	White	154	Marangani	c.629del	N	c.618	N	c.383	CC	c.354	N	c.265	N	c.259	N	c239-243ins	N	c.224-227del	N	c.92	TT	c.72	CC
Alpaca	White	156	Marangani	c.629del	Del	c.618	N	c.383	CC	c.354	N	c.265	N	c.259	N	c239-243ins	N	c.224-227del	N	c.92	TT	c.72	CC
Alpaca	White	158	Marangani	c.629del	Absent	c.618	AA	c.383	CC	c.354	CC	c.265	GG	c.259	GG	c239-243ins	Absent	c.224-227del	Absent	c.92	TT	c.72	CC
Alpaca	White	184	Marangani	c.629del	N	c.618	N	c.383	CC	c.354	N	c.265	N	c.259	N	c239-243ins	N	c.224-227del	N	c.92	TT	c.72	CC
Alpaca	White	186	Marangani	c.629del	N	c.618	N	c.383	CC	c.354	N	c.265	N	c.259	N	c239-243ins	N	c.224-227del	N	c.92	TT	c.72	CC
Alpaca	White	189	Marangani	c.629del	N	c.618	N	c.383	CC	c.354	N	c.265	N	c.259	N	c239-243ins	Homozygous	c.224-227del	N	c.92	TT	c.72	CC
Alpaca	White	191	Marangani	c.629del	N	c.618	N	c.383	CC	c.354	N	c.265	N	c.259	N	c239-243ins	N	c.224-227del	N	c.92	TT	c.72	CC
Alpaca	White	192	Marangani	c.629del	N	c.618	N	c.383	CC	c.354	N	c.265	N	c.259	N	c239-243ins	Homozygous	c.224-227del	N	c.92	TT	c.72	CC
Alpaca	White	201	Marangani	c.629del	N	c.618	GA	c.383	N	c.354	TC	c.265	N	c.259	AG	c239-243ins	N	c.224-227del	N	c.92	N	c.72	N
Alpaca	White	203	Marangani	c.629del	N	c.618	GG	c.383	N	c.354	TT	c.265	N	c.259	AA	c239-243ins	N	c.224-227del	N	c.92	N	c.72	N
Alpaca	White	205	Marangani	c.629del	N	c.618	GA	c.383	N	c.354	N	c.265	AG	c.259	N	c239-243ins	N	c.224-227del	N	c.92	N	c.72	GC
Alpaca	White	211	Marangani	c.629del	N	c.618	GA	c.383	N	c.354	TC	c.265	N	c.259	N	c239-243ins	N	c.224-227del	N	c.92	N	c.72	N
Alpaca	White	270	Marangani	c.629del	N	c.618	GA	c.383	N	c.354	TC	c.265	N	c.259	N	c239-243ins	N	c.224-227del	Heterozygous	c.92	N	c.72	N
Alpaca	White	274	La Raya	c.629del	N	c.618	GG	c.383	N	c.354	TT	c.265	N	c.259	AA	c239-243ins	N	c.224-227del	N	c.92	N	c.72	N
Alpaca	White	275	La Raya	c.629del	N	c.618	N	c.383	N	c.354	TC	c.265	N	c.259	N	c239-243ins	N	c.224-227del	N	c.92	N	c.72	N
Alpaca	White	276	La Raya	c.629del	N	c.618	GA	c.383	N	c.354	TC	c.265	N	c.259	N	c239-243ins	N	c.224-227del	Heterozygous	c.92	N	c.72	N
Alpaca	White	278	La Raya	c.629del	N	c.618	GA	c.383	N	c.354	TC	c.265	N	c.259	N	c239-243ins	N	c.224-227del	Heterozygous	c.92	N	c.72	N
Alpaca	White	282	La Raya	c.629del	N	c.618	GG	c.383	N	c.354	TT	c.265	N	c.259	AA	c239-243ins	N	c.224-227del	N	c.92	N	c.72	N
Alpaca	White	283	La Raya	c.629del	N	c.618	GG	c.383	N	c.354	TT	c.265	N	c.259	AG	c239-243ins	N	c.224-227del	Heterozygous	c.92	N	c.72	N
Alpaca	White	286	La Raya	c.629del	N	c.618	GA	c.383	TC	c.354	TC	c.265	N	c.259	N	c239-243ins	N	c.224-227del	N	c.92	N	c.72	N
Alpaca	White	287	La Raya	c.629del	N	c.618	N	c.383	N	c.354	N	c.265	N	c.259	N	c239-243ins	N	c.224-227del	N	c.92	N	c.72	N
Alpaca	White	291	La Raya	c.629del	N	c.618	GA	c.383	TC	c.354	TC	c.265	N	c.259	N	c239-243ins	N	c.224-227del	N	c.92	N	c.72	N
Alpaca	White	295	La Raya	c.629del	N	c.618	GA	c.383	N	c.354	TC	c.265	N	c.259	N	c239-243ins	N	c.224-227del	Heterozygous	c.92	N	c.72	N
Alpaca	White	344	La Raya	c.629del	N	c.618	GA	c.383	N	c.354	TC	c.265	N	c.259	N	c239-243ins	N	c.224-227del	Heterozygous	c.92	N	c.72	N
Alpaca	White	137	La Raya	c.629del	Absent	c.618	AA	c.383	CC	c.354	CC	c.265	GG	c.259	GG	c239-243ins	Absent	c.224-227del	Absent	c.92	TT	c.72	CC
Alpaca	White	138	La Raya	c.629del	N	c.618	GA	c.383	CC	c.354	TC	c.265	GG	c.259	GG	c239-243ins	Absent	c.224-227del	Heterozygous	c.92	TT	c.72	CC
Alpaca	White	139	La Raya	c.629del	N	c.618	N	c.383	CC	c.354	N	c.265	N	c.259	N	c239-243ins	N	c.224-227del	N	c.92	TT	c.72	CC
Alpaca	White	140	La Raya	c.629del	Absent	c.618	N	c.383	CC	c.354	TC	c.265	GG	c.259	GG	c239-243ins	Absent	c.224-227del	Heterozygous	c.92	TT	c.72	CC
Alpaca	White	141	La Raya	c.629del	Absent	c.618	AA	c.383	CC	c.354	CC	c.265	GG	c.259	GG	c239-243ins	Absent	c.224-227del	Absent	c.92	TT	c.72	CC
Alpaca	White	142	La Raya	c.629del	Absent	c.618	AA	c.383	CC	c.354	CC	c.265	GG	c.259	GG	c239-243ins	Absent	c.224-227del	Absent	c.92	TT	c.72	CC

Alpaca	White	143	La Raya	c.629del	Absent	c.618	AA	c.383	CC	c.354	CC	c.265	GG	c.259	GG	c239-243ins	Absent	c.224-227del	Absent	c.92	TT	c.72	CC
Alpaca	White	144	La Raya	c.629del	Absent	c.618	AA	c.383	CC	c.354	CC	c.265	GG	c.259	GG	c239-243ins	Absent	c.224-227del	Absent	c.92	TT	c.72	CC
Alpaca	White	145	La Raya	c.629del	Absent	c.618	AA	c.383	CC	c.354	CC	c.265	GG	c.259	GG	c239-243ins	Absent	c.224-227del	Absent	c.92	TT	c.72	CC
Alpaca	White	146	La Raya	c.629del	Absent	c.618	AA	c.383	CC	c.354	CC	c.265	GG	c.259	GG	c239-243ins	Absent	c.224-227del	Absent	c.92	TT	c.72	CC
Alpaca	White	147	La Raya	c.629del	N	c.618	N	c.383	CC	c.354	CC	c.265	GG	c.259	GG	c239-243ins	Absent	c.224-227del	Absent	c.92	N	c.72	N
Alpaca	White	148	Phinaya	c.629del	Absent	c.618	GA	c.383	CC	c.354	TC	c.265	GG	c.259	GG	c239-243ins	Absent	c.224-227del	Heterozygous	c.92	TT	c.72	CC
Alpaca	White	149	Phinaya	c.629del	Absent	c.618	AA	c.383	CC	c.354	CC	c.265	GG	c.259	GG	c239-243ins	Absent	c.224-227del	Absent	c.92	TT	c.72	CC
Alpaca	White	150	Phinaya	c.629del	Absent	c.618	AA	c.383	CC	c.354	CC	c.265	GG	c.259	N	c239-243ins	N	c.224-227del	Absent	c.92	N	c.72	N
Alpaca	White	151	Phinaya	c.629del	Absent	c.618	GA	c.383	TC	c.354	TC	c.265	GG	c.259	GG	c239-243ins	Absent	c.224-227del	Absent	c.92	TT	c.72	CC
Alpaca	White	158	Phinaya	c.629del	Absent	c.618	AA	c.383	CC	c.354	CC	c.265	GG	c.259	GG	c239-243ins	Absent	c.224-227del	Absent	c.92	TT	c.72	CC
Alpaca	White	159	Phinaya	c.629del	Absent	c.618	GA	c.383	CC	c.354	TC	c.265	GG	c.259	GG	c239-243ins	Absent	c.224-227del	Heterozygous	c.92	TT	c.72	CC
Alpaca	White	176	Phinaya	c.629del	Absent	c.618	GG	c.383	CC	c.354	TT	c.265	GG	c.259	AA	c239-243ins	Absent	c.224-227del	Absent	c.92	TT	c.72	CC
Alpaca	White	178	Phinaya	c.629del	Absent	c.618	GG	c.383	TC	c.354	TT	c.265	GG	c.259	AG	c239-243ins	Absent	c.224-227del	Absent	c.92	TT	c.72	CC
Alpaca	White	180	Phinaya	c.629del	Absent	c.618	GG	c.383	CC	c.354	TT	c.265	GG	c.259	AG	c239-243ins	Absent	c.224-227del	Heterozygous	c.92	TT	c.72	CC
Alpaca	White	181	Phinaya	c.629del	N	c.618	N	c.383	CC	c.354	N	c.265	N	c.259	N	c239-243ins	N	c.224-227del	N	c.92	N	c.72	N
Alpaca	White	182	Phinaya	c.629del	Absent	c.618	AA	c.383	CC	c.354	CC	c.265	GG	c.259	GG	c239-243ins	Absent	c.224-227del	Absent	c.92	TT	c.72	CC
Alpaca	White	184	Phinaya	c.629del	Absent	c.618	AA	c.383	CC	c.354	CC	c.265	GG	c.259	GG	c239-243ins	Absent	c.224-227del	Absent	c.92	TT	c.72	CC
Alpaca	White	188	Phinaya	c.629del	N	c.618	N	c.383	CC	c.354	CC	c.265	N	c.259	GG	c239-243ins	N	c.224-227del	Absent	c.92	N	c.72	N
Alpaca	White	193	Phinaya	c.629del	N	c.618	AA	c.383	CC	c.354	CC	c.265	N	c.259	GG	c239-243ins	N	c.224-227del	Absent	c.92	N	c.72	N
Alpaca	White	194	Phinaya	c.629del	N	c.618	N	c.383	CC	c.354	TC	c.265	N	c.259	GG	c239-243ins	N	c.224-227del	Absent	c.92	N	c.72	N
Alpaca	White	106	Phinaya	c.629del	N	c.618	AA	c.383	CC	c.354	CC	c.265	N	c.259	GG	c239-243ins	N	c.224-227del	Absent	c.92	N	c.72	N
Alpaca	White	107	Phinaya	c.629del	N	c.618	GA	c.383	TC	c.354	TC	c.265	N	c.259	GG	c239-243ins	N	c.224-227del	Absent	c.92	N	c.72	N
Alpaca	White	113	Phinaya	c.629del	N	c.618	AA	c.383	CC	c.354	CC	c.265	N	c.259	GG	c239-243ins	N	c.224-227del	Absent	c.92	N	c.72	N
Alpaca	White	117	Phinaya	c.629del	Absent	c.618	GA	c.383	TC	c.354	TC	c.265	GG	c.259	GG	c239-243ins	Absent	c.224-227del	Absent	c.92	TT	c.72	CC
Alpaca	White	120	Phinaya	c.629del	Absent	c.618	N	c.383	CC	c.354	CC	c.265	GG	c.259	GG	c239-243ins	Absent	c.224-227del	Absent	c.92	TT	c.72	CC
Alpaca	White	122	Phinaya	c.629del	Absent	c.618	AA	c.383	CC	c.354	CC	c.265	GG	c.259	GG	c239-243ins	Absent	c.224-227del	Absent	c.92	TT	c.72	CC
Alpaca	White	126	Phinaya	c.629del	Absent	c.618	GA	c.383	CC	c.354	TC	c.265	GG	c.259	GG	c239-243ins	Absent	c.224-227del	Heterozygous	c.92	TT	c.72	CC
Alpaca	White	132	Phinaya	c.629del	Absent	c.618	AA	c.383	CC	c.354	CC	c.265	GG	c.259	GG	c239-243ins	Absent	c.224-227del	Absent	c.92	TT	c.72	CC
Alpaca	White	136	Phinaya	c.629del	Absent	c.618	GA	c.383	TC	c.354	TC	c.265	GG	c.259	GG	c239-243ins	Absent	c.224-227del	Absent	c.92	TT	c.72	CC
Alpaca	White	139	Phinaya	c.629del	Absent	c.618	AA	c.383	CC	c.354	CC	c.265	GG	c.259	GG	c239-243ins	Absent	c.224-227del	Absent	c.92	TT	c.72	CC
Alpaca	White	140	Phinaya	c.629del	Absent	c.618	GA	c.383	CC	c.354	TC	c.265	N	c.259	GG	c239-243ins	Absent	c.224-227del	Heterozygous	c.92	TT	c.72	CC
Alpaca	White	143	Phinaya	c.629del	Absent	c.618	AA	c.383	CC	c.354	CC	c.265	GG	c.259	GG	c239-243ins	Absent	c.224-227del	Absent	c.92	TT	c.72	CC
Alpaca	White	146	Phinaya	c.629del	Absent	c.618	AA	c.383	CC	c.354	CC	c.265	GG	c.259	GG	c239-243ins	Absent	c.224-227del	Absent	c.92	TT	c.72	CC
Alpaca	White	147	Phinaya	c.629del	Absent	c.618	AA	c.383	CC	c.354	CC	c.265	GG	c.259	GG	c239-243ins	Absent	c.224-227del	Absent	c.92	TT	c.72	CC
Alpaca	White	149	Phinaya	c.629del	Absent	c.618	GA	c.383	CC	c.354	TC	c.265	GG	c.259	GG	c239-243ins	Absent	c.224-227del	Heterozygous	c.92	TT	c.72	CC
Alpaca	White	152	Phinaya	c.629del	Absent	c.618	GA	c.383	TC	c.354	TC	c.265	GG	c.259	GG	c239-243ins	Absent	c.224-227del	Absent	c.92	TT	c.72	CC
Alpaca	White	153	Phinaya	c.629del	Absent	c.618	AA	c.383	CC	c.354	CC	c.265	GG	c.259	GG	c239-243ins	Absent	c.224-227del	Absent	c.92	TT	c.72	CC
Alpaca	White	154	Nuñoa	c.629del	Absent	c.618	AA	c.383	CC	c.354	CC	c.265	GG	c.259	GG	c239-243ins	Absent	c.224-227del	Absent	c.92	TT	c.72	CC
Alpaca	White	155	Nuñoa	c.629del	Absent	c.618	AA	c.383	CC	c.354	CC	c.265	GG	c.259	GG	c239-243ins	Absent	c.224-227del	Absent	c.92	TT	c.72	CC
Alpaca	White	156	Nuñoa	c.629del	Absent	c.618	GA	c.383	CC	c.354	TC	c.265	GG	c.259	GG	c239-243ins	Absent	c.224-227del	Heterozygous	c.92	TT	c.72	CC
Alpaca	White	157	Nuñoa	c.629del	Absent	c.618	AA	c.383	CC	c.354	CC	c.265	GG	c.259	GG	c239-243ins	Absent	c.224-227del	Absent	c.92	TT	c.72	CC
Alpaca	White	158	Nuñoa	c.629del	Absent	c.618	AA	c.383	CC	c.354	CC	c.265	GG	c.259	GG	c239-243ins	Absent	c.224-227del	Absent	c.92	TT	c.72	CC
Alpaca	White	159	Nuñoa	c.629del	Absent	c.618	AA	c.383	CC	c.354	CC	c.265	GG	c.259	GG	c239-243ins	Absent	c.224-227del	Absent	c.92	TT	c.72	CC
Alpaca	White	160	Nuñoa	c.629del	Absent	c.618	AA	c.383	CC	c.354	CC	c.265	GG	c.259	GG	c239-243ins	Absent	c.224-227del	Absent	c.92	TT	c.72	CC
Alpaca	White	161	Nuñoa	c.629del	Absent	c.618	AA	c.383	CC	c.354	CC	c.265	GG	c.259	GG	c239-243ins	Absent	c.224-227del	Absent	c.92	TT	c.72	CC
Alpaca	White	162	Nuñoa	c.629del	Absent	c.618	GA	c.383	CC	c.354	TC	c.265	GG	c.259	AG	c239-243ins	Absent	c.224-227del	Absent	c.92	TT	c.72	CC
Alpaca	White	164	Nuñoa	c.629del	Absent	c.618	AA	c.383	CC	c.354	CC	c.265	GG	c.259	GG	c239-243ins	Absent	c.224-227del	Absent	c.92	TT	c.72	CC

Alpaca	White	134	Nuñoa	c.629del	Absent	c.618	AA	c.383	CC	c.354	TC	c.265	GG	c.259	GG	c239-243ins	Absent	c.224-227del	Absent	c.92	TT	c.72	CC
Alpaca	White	137	Nuñoa	c.629del	Absent	c.618	AA	c.383	CC	c.354	CC	c.265	GG	c.259	GG	c239-243ins	Absent	c.224-227del	Absent	c.92	TT	c.72	CC
Alpaca	White	138	Nuñoa	c.629del	Absent	c.618	AA	c.383	CC	c.354	CC	c.265	GG	c.259	GG	c239-243ins	Absent	c.224-227del	Absent	c.92	TT	c.72	CC
Alpaca	White	139	Nuñoa	c.629del	Absent	c.618	AA	c.383	CC	c.354	CC	c.265	GG	c.259	GG	c239-243ins	Absent	c.224-227del	Absent	c.92	N	c.72	N
Alpaca	White	121	Nuñoa	c.629del	Absent	c.618	GG	c.383	CC	c.354	TT	c.265	GG	c.259	AA	c239-243ins	Absent	c.224-227del	Absent	c.92	TT	c.72	CC
Alpaca	White	122	Nuñoa	c.629del	Absent	c.618	AA	c.383	CC	c.354	CC	c.265	GG	c.259	GG	c239-243ins	Absent	c.224-227del	Absent	c.92	TT	c.72	CC
Alpaca	White	123	Nuñoa	c.629del	Absent	c.618	AA	c.383	CC	c.354	CC	c.265	GG	c.259	GG	c239-243ins	Absent	c.224-227del	Absent	c.92	TT	c.72	CC
Alpaca	White	126	Nuñoa	c.629del	Absent	c.618	AA	c.383	CC	c.354	CC	c.265	GG	c.259	GG	c239-243ins	Absent	c.224-227del	Absent	c.92	TT	c.72	CC
Alpaca	White	128	Nuñoa	c.629del	N	c.618	N	c.383	CC	c.354	N	c.265	N	c.259	N	c239-243ins	N	c.224-227del	Absent	c.92	TT	c.72	CC
Alpaca	White	132	Nuñoa	c.629del	Absent	c.618	AA	c.383	CC	c.354	CC	c.265	GG	c.259	GG	c239-243ins	Absent	c.224-227del	Absent	c.92	TT	c.72	CC
Alpaca	White	146	Nuñoa	c.629del	Absent	c.618	AA	c.383	CC	c.354	CC	c.265	GG	c.259	GG	c239-243ins	Absent	c.224-227del	Absent	c.92	TT	c.72	CC
Alpaca	White	147	Nuñoa	c.629del	Absent	c.618	GG	c.383	TC	c.354	TT	c.265	GG	c.259	GG	c239-243ins	Absent	c.224-227del	Heterozygous	c.92	TT	c.72	CC
Alpaca	White	148	Nuñoa	c.629del	Absent	c.618	GG	c.383	TC	c.354	TT	c.265	GG	c.259	GG	c239-243ins	N	c.224-227del	Heterozygous	c.92	TT	c.72	CC
Alpaca	White	152	Nuñoa	c.629del	Absent	c.618	GG	c.383	CC	c.354	TT	c.265	GG	c.259	AA	c239-243ins	Absent	c.224-227del	Absent	c.92	TT	c.72	CC
Alpaca	White	154	Nuñoa	c.629del	N	c.618	N	c.383	CC	c.354	N	c.265	N	c.259	N	c239-243ins	N	c.224-227del	N	c.92	TT	c.72	CC
Alpaca	White	156	Nuñoa	c.629del	Del	c.618	N	c.383	CC	c.354	N	c.265	N	c.259	N	c239-243ins	N	c.224-227del	N	c.92	TT	c.72	CC
Alpaca	White	109	Nuñoa	c.629del	Absent	c.618	AA	c.383	CC	c.354	CC	c.265	GG	c.259	GG	c239-243ins	Absent	c.224-227del	Absent	c.92	N	c.72	CC
Alpaca	White	112	Nuñoa	c.629del	Absent	c.618	AA	c.383	CC	c.354	CC	c.265	GG	c.259	GG	c239-243ins	Absent	c.224-227del	Absent	c.92	TT	c.72	CC
Alpaca	White	113	Nuñoa	c.629del	Absent	c.618	AA	c.383	CC	c.354	CC	c.265	GG	c.259	GG	c239-243ins	Absent	c.224-227del	Absent	c.92	TT	c.72	CC
Alpaca	White	114	Nuñoa	c.629del	Absent	c.618	AA	c.383	CC	c.354	CC	c.265	GG	c.259	GG	c239-243ins	Absent	c.224-227del	Absent	c.92	TT	c.72	CC
Alpaca	White	115	Nuñoa	c.629del	Absent	c.618	N	c.383	CC	c.354	CC	c.265	GG	c.259	GG	c239-243ins	Absent	c.224-227del	Absent	c.92	TT	c.72	CC
Alpaca	White	120	Nuñoa	c.629del	Absent	c.618	AA	c.383	CC	c.354	TC	c.265	GG	c.259	GG	c239-243ins	Absent	c.224-227del	Absent	c.92	TT	c.72	CC
Alpaca	White	158	Nuñoa	c.629del	Absent	c.618	AA	c.383	CC	c.354	CC	c.265	GG	c.259	GG	c239-243ins	Absent	c.224-227del	Absent	c.92	TT	c.72	CC
Alpaca	White	164	Nuñoa	c.629del	Absent	c.618	AA	c.383	CC	c.354	CC	c.265	GG	c.259	GG	c239-243ins	Absent	c.224-227del	Absent	c.92	N	c.72	N
Alpaca	White	167	Nuñoa	c.629del	N	c.618	N	c.383	CC	c.354	N	c.265	N	c.259	N	c239-243ins	Homozygous	c.224-227del	N	c.92	TT	c.72	CC
Alpaca	White	171	Nuñoa	c.629del	N	c.618	N	c.383	CC	c.354	CC	c.265	N	c.259	N	c239-243ins	N	c.224-227del	N	c.92	N	c.72	N
Alpaca	White	177	Nuñoa	c.629del	N	c.618	N	c.383	CC	c.354	N	c.265	N	c.259	N	c239-243ins	N	c.224-227del	N	c.92	TT	c.72	CC
Alpaca	White	127	Nuñoa	c.629del	N	c.618	N	c.383	CC	c.354	N	c.265	N	c.259	N	c239-243ins	Heterozygous	c.224-227del	N	c.92	TT	c.72	CC
Alpaca	White	129	Nuñoa	c.629del	Absent	c.618	GA	c.383	CC	c.354	TC	c.265	GG	c.259	GG	c239-243ins	Absent	c.224-227del	Heterozygous	c.92	TT	c.72	CC
Alpaca	White	130	Nuñoa	c.629del	Absent	c.618	GA	c.383	CC	c.354	TC	c.265	GG	c.259	GG	c239-243ins	Absent	c.224-227del	Heterozygous	c.92	TT	c.72	CC
Alpaca	White	131	Nuñoa	c.629del	Absent	c.618	AA	c.383	CC	c.354	CC	c.265	GG	c.259	GG	c239-243ins	Absent	c.224-227del	Absent	c.92	TT	c.72	CC
Alpaca	White	133	Nuñoa	c.629del	Absent	c.618	GA	c.383	CC	c.354	TC	c.265	GG	c.259	GG	c239-243ins	Absent	c.224-227del	Heterozygous	c.92	TT	c.72	CC
Alpaca	White	134	Nuñoa	c.629del	Absent	c.618	AA	c.383	CC	c.354	CC	c.265	GG	c.259	GG	c239-243ins	Absent	c.224-227del	Absent	c.92	TT	c.72	CC
Alpaca	White	184	Nuñoa	c.629del	N	c.618	N	c.383	CC	c.354	N	c.265	N	c.259	N	c239-243ins	N	c.224-227del	N	c.92	TT	c.72	CC
Alpaca	White	186	Nuñoa	c.629del	N	c.618	N	c.383	CC	c.354	N	c.265	N	c.259	N	c239-243ins	N	c.224-227del	N	c.92	TT	c.72	CC
Alpaca	White	187	Nuñoa	c.629del	Absent	c.618	AA	c.383	CC	c.354	CC	c.265	GG	c.259	GG	c239-243ins	Absent	c.224-227del	Absent	c.92	TT	c.72	CC
Alpaca	White	189	Nuñoa	c.629del	N	c.618	N	c.383	CC	c.354	N	c.265	N	c.259	N	c239-243ins	Homozygous	c.224-227del	N	c.92	TT	c.72	CC
Alpaca	White	191	Nuñoa	c.629del	N	c.618	N	c.383	CC	c.354	N	c.265	N	c.259	N	c239-243ins	N	c.224-227del	N	c.92	TT	c.72	CC
Alpaca	White	192	Nuñoa	c.629del	N	c.618	N	c.383	CC	c.354	N	c.265	N	c.259	N	c239-243ins	Homozygous	c.224-227del	N	c.92	TT	c.72	CC
Alpaca	White	137	Macusani	c.629del	Absent	c.618	N	c.383	CC	c.354	CC	c.265	GG	c.259	GG	c239-243ins	Absent	c.224-227del	Absent	c.92	N	c.72	N
Alpaca	White	142	Macusani	c.629del	Absent	c.618	GA	c.383	CC	c.354	TC	c.265	N	c.259	GG	c239-243ins	Absent	c.224-227del	Heterozygous	c.92	N	c.72	N
Alpaca	White	143	Macusani	c.629del	Absent	c.618	GA	c.383	CC	c.354	TC	c.265	GG	c.259	GG	c239-243ins	Absent	c.224-227del	Heterozygous	c.92	N	c.72	N
Alpaca	White	146	Macusani	c.629del	N	c.618	N	c.383	CC	c.354	N	c.265	N	c.259	N	c239-243ins	Heterozygous	c.224-227del	N	c.92	TT	c.72	CC
Alpaca	White	147	Macusani	c.629del	N	c.618	N	c.383	CC	c.354	N	c.265	N	c.259	N	c239-243ins	Homozygous	c.224-227del	Absent	c.92	TT	c.72	CC
Alpaca	White	148	Macusani	c.629del	N	c.618	N	c.383	CC	c.354	N	c.265	N	c.259	N	c239-243ins	N	c.224-227del	N	c.92	TT	c.72	CC
Alpaca	White	149	Macusani	c.629del	Absent	c.618	AA	c.383	CC	c.354	N	c.265	GG	c.259	GG	c239-243ins	Absent	c.224-227del	Absent	c.92	TT	c.72	N
Alpaca	White	152	Macusani	c.629del	Absent	c.618	AA	c.383	CC	c.354	N	c.265	GG	c.259	GG	c239-243ins	Absent	c.224-227del	Absent	c.92	TT	c.72	N

Alpaca	White	157	Macusani	c.629del	Absent	c.618	AA	c.383	CC	c.354	CC	c.265	GG	c.259	GG	c239-243ins	Absent	c.224-227del	Absent	c.92	TT	c.72	N
Alpaca	White	160	Macusani	c.629del	Absent	c.618	GA	c.383	CC	c.354	N	c.265	GG	c.259	N	c239-243ins	Absent	c.224-227del	Absent	c.92	TT	c.72	N
Alpaca	White	222	Macusani	c.629del	Absent	c.618	AA	c.383	CC	c.354	CC	c.265	GG	c.259	GG	c239-243ins	Absent	c.224-227del	Absent	c.92	TT	c.72	CC
Alpaca	White	223	Macusani	c.629del	Absent	c.618	AA	c.383	CC	c.354	CC	c.265	GG	c.259	GG	c239-243ins	Absent	c.224-227del	Absent	c.92	TT	c.72	CC
Alpaca	White	224	Macusani	c.629del	Absent	c.618	AA	c.383	CC	c.354	CC	c.265	GG	c.259	GG	c239-243ins	Absent	c.224-227del	Absent	c.92	TT	c.72	CC
Alpaca	White	225	Macusani	c.629del	Absent	c.618	AA	c.383	CC	c.354	CC	c.265	GG	c.259	GG	c239-243ins	Absent	c.224-227del	Absent	c.92	TT	c.72	CC
Alpaca	White	226	Macusani	c.629del	N	c.618	N	c.383	CC	c.354	CC	c.265	N	c.259	N	c239-243ins	Homozygous	c.224-227del	Absent	c.92	TT	c.72	CC
Alpaca	White	227	Macusani	c.629del	N	c.618	N	c.383	CC	c.354	N	c.265	N	c.259	N	c239-243ins	Homozygous	c.224-227del	Absent	c.92	TT	c.72	CC
Alpaca	White	228	Macusani	c.629del	N	c.618	N	c.383	CC	c.354	N	c.265	N	c.259	N	c239-243ins	Homozygous	c.224-227del	Absent	c.92	TT	c.72	CC
Alpaca	White	229	Macusani	c.629del	N	c.618	N	c.383	CC	c.354	N	c.265	N	c.259	N	c239-243ins	N	c.224-227del	Absent	c.92	TT	c.72	CC
Alpaca	White	230	Macusani	c.629del	N	c.618	N	c.383	CC	c.354	N	c.265	N	c.259	N	c239-243ins	N	c.224-227del	N	c.92	TT	c.72	CC
Alpaca	White	231	Macusani	c.629del	Absent	c.618	AA	c.383	CC	c.354	CC	c.265	GG	c.259	GG	c239-243ins	Absent	c.224-227del	Absent	c.92	TT	c.72	CC
Alpaca	White	232	Macusani	c.629del	N	c.618	N	c.383	CC	c.354	N	c.265	N	c.259	N	c239-243ins	Heterozygous	c.224-227del	N	c.92	TT	c.72	CC
Alpaca	Brown	154	Marangani	c.629del	Absent	c.618	GA	c.383	TC	c.354	TC	c.265	GG	c.259	AG	c239-243ins	Absent	c.224-227del	Absent	c.92	TT	c.72	CC
Alpaca	Brown	155	Marangani	c.629del	N	c.618	N	c.383	CC	c.354	N	c.265	N	c.259	N	c239-243ins	N	c.224-227del	N	c.92	TT	c.72	CC
Alpaca	Brown	156	Marangani	c.629del	Absent	c.618	GA	c.383	CC	c.354	TC	c.265	GG	c.259	AG	c239-243ins	Absent	c.224-227del	Absent	c.92	TT	c.72	CC
Alpaca	Brown	157	Marangani	c.629del	Absent	c.618	GA	c.383	CC	c.354	TC	c.265	GG	c.259	GG	c239-243ins	Absent	c.224-227del	Absent	c.92	CT	c.72	CC
Alpaca	Brown	174	Marangani	c.629del	Absent	c.618	AA	c.383	CC	c.354	CC	c.265	GG	c.259	GG	c239-243ins	Absent	c.224-227del	Absent	c.92	TT	c.72	CC
Alpaca	Brown	175	Marangani	c.629del	Absent	c.618	AA	c.383	CC	c.354	CC	c.265	GG	c.259	GG	c239-243ins	Absent	c.224-227del	Absent	c.92	TT	c.72	CC
Alpaca	Brown	177	Marangani	c.629del	Absent	c.618	AA	c.383	CC	c.354	CC	c.265	GG	c.259	GG	c239-243ins	Absent	c.224-227del	Absent	c.92	N	c.72	N
Alpaca	Brown	102	Marangani	c.629del	N	c.618	GG	c.383	CC	c.354	TT	c.265	N	c.259	AA	c239-243ins	N	c.224-227del	Absent	c.92	N	c.72	N
Alpaca	Brown	103	Marangani	c.629del	N	c.618	GA	c.383	CC	c.354	TC	c.265	N	c.259	AG	c239-243ins	N	c.224-227del	Absent	c.92	N	c.72	N
Alpaca	Brown	104	Marangani	c.629del	N	c.618	GA	c.383	CC	c.354	TC	c.265	N	c.259	AG	c239-243ins	N	c.224-227del	Absent	c.92	N	c.72	N
Alpaca	Brown	105	La Raya	c.629del	N	c.618	GG	c.383	CC	c.354	TT	c.265	N	c.259	AA	c239-243ins	N	c.224-227del	Absent	c.92	N	c.72	N
Alpaca	Brown	111	La Raya	c.629del	N	c.618	GA	c.383	CC	c.354	TC	c.265	N	c.259	AG	c239-243ins	N	c.224-227del	Absent	c.92	N	c.72	N
Alpaca	Brown	114	La Raya	c.629del	N	c.618	GA	c.383	CC	c.354	TC	c.265	N	c.259	AG	c239-243ins	N	c.224-227del	Absent	c.92	N	c.72	N
Alpaca	Brown	118	La Raya	c.629del	Absent	c.618	GA	c.383	CC	c.354	TC	c.265	GG	c.259	AG	c239-243ins	Absent	c.224-227del	Absent	c.92	TT	c.72	CC
Alpaca	Brown	121	La Raya	c.629del	Absent	c.618	GA	c.383	CC	c.354	TC	c.265	GG	c.259	AG	c239-243ins	Absent	c.224-227del	Absent	c.92	TT	c.72	CC
Alpaca	Brown	123	La Raya	c.629del	N	c.618	N	c.383	CC	c.354	N	c.265	N	c.259	N	c239-243ins	Homozygous	c.224-227del	N	c.92	TT	c.72	CC
Alpaca	Brown	125	La Raya	c.629del	Absent	c.618	GA	c.383	CC	c.354	TC	c.265	GG	c.259	AG	c239-243ins	Absent	c.224-227del	Absent	c.92	TT	c.72	CC
Alpaca	Brown	127	La Raya	c.629del	Absent	c.618	GA	c.383	TC	c.354	TC	c.265	GG	c.259	GG	c239-243ins	Absent	c.224-227del	Absent	c.92	TT	c.72	CC
Alpaca	Brown	128	La Raya	c.629del	Absent	c.618	GG	c.383	TC	c.354	TT	c.265	GG	c.259	AG	c239-243ins	Absent	c.224-227del	Absent	c.92	TT	c.72	CC
Alpaca	Brown	129	La Raya	c.629del	Absent	c.618	GG	c.383	TC	c.354	TT	c.265	GG	c.259	AG	c239-243ins	Absent	c.224-227del	Absent	c.92	TT	c.72	CC
Alpaca	Brown	130	Phinaya	c.629del	N	c.618	GA	c.383	CC	c.354	TC	c.265	GG	c.259	AG	c239-243ins	Absent	c.224-227del	Absent	c.92	TT	c.72	CC
Alpaca	Brown	133	Phinaya	c.629del	Absent	c.618	GA	c.383	CC	c.354	TC	c.265	GG	c.259	AG	c239-243ins	Absent	c.224-227del	Absent	c.92	TT	c.72	CC
Alpaca	Brown	137	Phinaya	c.629del	Absent	c.618	GA	c.383	CC	c.354	TC	c.265	GG	c.259	AG	c239-243ins	Absent	c.224-227del	Absent	c.92	TT	c.72	CC
Alpaca	Brown	145	Phinaya	c.629del	Absent	c.618	GA	c.383	CC	c.354	TC	c.265	GG	c.259	AG	c239-243ins	Absent	c.224-227del	Absent	c.92	TT	c.72	CC
Alpaca	Brown	150	Phinaya	c.629del	Absent	c.618	GA	c.383	CC	c.354	TC	c.265	GG	c.259	AG	c239-243ins	Absent	c.224-227del	Absent	c.92	TT	c.72	CC
Alpaca	Brown	151	Phinaya	c.629del	Absent	c.618	GG	c.383	CC	c.354	TT	c.265	GG	c.259	AA	c239-243ins	Absent	c.224-227del	Absent	c.92	TT	c.72	CC
Alpaca	Brown	163	Phinaya	c.629del	N	c.618	N	c.383	CC	c.354	N	c.265	N	c.259	N	c239-243ins	Homozygous	c.224-227del	Absent	c.92	TT	c.72	CC
Alpaca	Brown	140	Phinaya	c.629del	Absent	c.618	GG	c.383	CC	c.354	TT	c.265	GG	c.259	AA	c239-243ins	Absent	c.224-227del	Absent	c.92	N	c.72	N
Alpaca	Brown	143	Macusani	c.629del	Absent	c.618	GG	c.383	TC	c.354	TT	c.265	GG	c.259	GG	c239-243ins	Absent	c.224-227del	Heterozygous	c.92	TT	c.72	CC
Alpaca	Brown	124	Macusani	c.629del	Absent	c.618	AA	c.383	CC	c.354	CC	c.265	GG	c.259	GG	c239-243ins	Absent	c.224-227del	Absent	c.92	TT	c.72	CC
Alpaca	Brown	125	Macusani	c.629del	Absent	c.618	AA	c.383	CC	c.354	CC	c.265	GG	c.259	GG	c239-243ins	Absent	c.224-227del	Absent	c.92	TT	c.72	CC
Alpaca	Brown	110	Nuñoa	c.629del	Absent	c.618	GG	c.383	CC	c.354	TT	c.265	GG	c.259	AA	c239-243ins	Absent	c.224-227del	Absent	c.92	TT	c.72	CC
Alpaca	Brown	135	Nuñoa	c.629del	Heterozygous	c.618	N	c.383	CC	c.354	TC	c.265	GG	c.259	AG	c239-243ins	Absent	c.224-227del	Absent	c.92	TT	c.72	CC
Alpaca	Brown	185	Nuñoa	c.629del	N	c.618	N	c.383	CC	c.354	N	c.265	N	c.259	N	c239-243ins	N	c.224-227del	N	c.92	TT	c.72	CC

Alpaca	Brown	188	Nuñoa	c.629del	Absent	c.618	GA	c.383	CC	c.354	TC	c.265	GG	c.259	AG	c239-243ins	Absent	c.224-227del	Absent	c.92	TT	c.72	CC
Alpaca	Brown	150	Nuñoa	c.629del	Heterozygous	c.618	N	c.383	CC	c.354	TT	c.265	GG	c.259	AA	c239-243ins	N	c.224-227del	Absent	c.92	TT	c.72	CC
Alpaca	Brown	154	Nuñoa	c.629del	Del	c.618	N	c.383	N	c.354	TT	c.265	GG	c.259	AA	c239-243ins	N	c.224-227del	Absent	c.92	TT	c.72	GC
Alpaca	Brown	155	Nuñoa	c.629del	Del	c.618	N	c.383	CC	c.354	TC	c.265	AG	c.259	AA	c239-243ins	Heterozygous	c.224-227del	Absent	c.92	TT	c.72	CC
Alpaca	Brown	201	Nuñoa	c.629del	Heterozygous	c.618	N	c.383	CC	c.354	TC	c.265	GG	c.259	AG	c239-243ins	Heterozygous	c.224-227del	Absent	c.92	TT	c.72	CC
Alpaca	Brown	203	Nuñoa	c.629del	Del	c.618	N	c.383	CC	c.354	TT	c.265	GG	c.259	AA	c239-243ins	Heterozygous	c.224-227del	Absent	c.92	TT	c.72	GC
Alpaca	Brown	204	Nuñoa	c.629del	Del	c.618	N	c.383	CC	c.354	TT	c.265	GG	c.259	AA	c239-243ins	Homozygous	c.224-227del	Absent	c.92	TT	c.72	CC
Alpaca	Brown	205	Nuñoa	c.629del	Heterozygous	c.618	N	c.383	CC	c.354	TT	c.265	AG	c.259	AA	c239-243ins	Absent	c.224-227del	Absent	c.92	TT	c.72	CC
Alpaca	Brown	206	Nuñoa	c.629del	Del	c.618	N	c.383	CC	c.354	TT	c.265	GG	c.259	AA	c239-243ins	Homozygous	c.224-227del	Absent	c.92	TT	c.72	CC
Alpaca	Brown	210	Nuñoa	c.629del	Heterozygous	c.618	N	c.383	CC	c.354	TC	c.265	GG	c.259	AG	c239-243ins	Heterozygous	c.224-227del	Absent	c.92	TT	c.72	CC
Alpaca	Brown	211	Nuñoa	c.629del	Del	c.618	N	c.383	CC	c.354	TT	c.265	GG	c.259	AA	c239-243ins	Heterozygous	c.224-227del	Absent	c.92	TT	c.72	GC
Alpaca	Brown	213	Nuñoa	c.629del	Del	c.618	N	c.383	N	c.354	TC	c.265	GG	c.259	AA	c239-243ins	Heterozygous	c.224-227del	Absent	c.92	TT	c.72	CC
Alpaca	Brown	215	Nuñoa	c.629del	Del	c.618	N	c.383	CC	c.354	TT	c.265	AG	c.259	AA	c239-243ins	Heterozygous	c.224-227del	Absent	c.92	TT	c.72	CC
Alpaca	Brown	218	Nuñoa	c.629del	Heterozygous	c.618	N	c.383	CC	c.354	TC	c.265	GG	c.259	AG	c239-243ins	Absent	c.224-227del	Absent	c.92	TT	c.72	GC
Alpaca	Brown	219	Nuñoa	c.629del	Del	c.618	N	c.383	CC	c.354	TT	c.265	GG	c.259	AA	c239-243ins	Heterozygous	c.224-227del	Absent	c.92	TT	c.72	GC
Alpaca	Brown	244	Nuñoa	c.629del	Del	c.618	N	c.383	CC	c.354	TT	c.265	GG	c.259	AA	c239-243ins	Homozygous	c.224-227del	Absent	c.92	TT	c.72	CC
Alpaca	Brown	241	Nuñoa	c.629del	N	c.618	N	c.383	N	c.354	N	c.265	GG	c.259	N	c239-243ins	N	c.224-227del	N	c.92	N	c.72	N
Alpaca	LF	152	Marangani	c.629del	Absent	c.618	GG	c.383	CC	c.354	TT	c.265	GG	c.259	GG	c239-243ins	Absent	c.224-227del	Homozygous	c.92	N	c.72	N
Alpaca	LF	153	Marangani	c.629del	Absent	c.618	GA	c.383	CC	c.354	TC	c.265	GG	c.259	AG	c239-243ins	Absent	c.224-227del	Absent	c.92	N	c.72	N
Alpaca	LF	173	Phinaya	c.629del	Absent	c.618	GA	c.383	CC	c.354	TC	c.265	GG	c.259	GG	c239-243ins	Absent	c.224-227del	Heterozygous	c.92	TT	c.72	CC
Alpaca	LF	179	Phinaya	c.629del	Absent	c.618	GA	c.383	CC	c.354	TC	c.265	GG	c.259	AG	c239-243ins	Absent	c.224-227del	Absent	c.92	TT	c.72	CC
Alpaca	LF	190	Nuñoa	c.629del	N	c.618	AA	c.383	CC	c.354	CC	c.265	N	c.259	GG	c239-243ins	N	c.224-227del	Absent	c.92	N	c.72	N
Alpaca	LF	112	Nuñoa	c.629del	N	c.618	GA	c.383	CC	c.354	TC	c.265	N	c.259	GG	c239-243ins	N	c.224-227del	Heterozygous	c.92	N	c.72	N
Alpaca	LF	131	Nuñoa	c.629del	Absent	c.618	AA	c.383	CC	c.354	CC	c.265	GG	c.259	GG	c239-243ins	Absent	c.224-227del	Absent	c.92	TT	c.72	CC
Alpaca	LF	135	Nuñoa	c.629del	Absent	c.618	GA	c.383	CC	c.354	TC	c.265	GG	c.259	AG	c239-243ins	Absent	c.224-227del	Absent	c.92	TT	c.72	CC
Alpaca	LF	138	Nuñoa	c.629del	Absent	c.618	AA	c.383	CC	c.354	CC	c.265	GG	c.259	GG	c239-243ins	Absent	c.224-227del	Absent	c.92	TT	c.72	CC
Alpaca	LF	127	La Raya	c.629del	Absent	c.618	AA	c.383	CC	c.354	CC	c.265	GG	c.259	GG	c239-243ins	Absent	c.224-227del	Absent	c.92	TT	c.72	CC
Alpaca	LF	133	La Raya	c.629del	Absent	c.618	AA	c.383	CC	c.354	CC	c.265	GG	c.259	GG	c239-243ins	Absent	c.224-227del	Absent	c.92	TT	c.72	CC
Alpaca	LF	150	La Raya	c.629del	Absent	c.618	GA	c.383	CC	c.354	TC	c.265	GG	c.259	GG	c239-243ins	Absent	c.224-227del	Heterozygous	c.92	TT	c.72	CC
Alpaca	LF	136	La Raya	c.629del	Absent	c.618	GA	c.383	CC	c.354	TC	c.265	GG	c.259	GG	c239-243ins	Absent	c.224-227del	Heterozygous	c.92	TT	c.72	CC
Alpaca	LF	208	La Raya	c.629del	Absent	c.618	GG	c.383	CC	c.354	TT	c.265	GG	c.259	GG	c239-243ins	Absent	c.224-227del	Homozygous	c.92	N	c.72	N
Alpaca	Black	183	Marangani	c.629del	N	c.618	GA	c.383	N	c.354	N	c.265	AG	c.259	N	c239-243ins	N	c.224-227del	N	c.92	N	c.72	GC
Alpaca	Black	186	Marangani	c.629del	N	c.618	GA	c.383	N	c.354	TC	c.265	N	c.259	N	c239-243ins	N	c.224-227del	Heterozygous	c.92	N	c.72	N
Alpaca	Black	195	Marangani	c.629del	N	c.618	GG	c.383	N	c.354	TT	c.265	N	c.259	AA	c239-243ins	N	c.224-227del	N	c.92	N	c.72	N
Alpaca	Black	196	Marangani	c.629del	N	c.618	GA	c.383	N	c.354	TC	c.265	N	c.259	AG	c239-243ins	N	c.224-227del	N	c.92	N	c.72	N
Alpaca	Black	134	Marangani	c.629del	N	c.618	GG	c.383	N	c.354	TT	c.265	N	c.259	AA	c239-243ins	N	c.224-227del	N	c.92	N	c.72	N
Alpaca	Black	141	Marangani	c.629del	N	c.618	GG	c.383	N	c.354	TT	c.265	N	c.259	AA	c239-243ins	N	c.224-227del	N	c.92	N	c.72	N
Alpaca	Black	142	Marangani	c.629del	N	c.618	GA	c.383	N	c.354	TT	c.265	N	c.259	AG	c239-243ins	N	c.224-227del	N	c.92	N	c.72	N
Alpaca	Black	144	Marangani	c.629del	N	c.618	GG	c.383	TC	c.354	TT	c.265	N	c.259	AG	c239-243ins	N	c.224-227del	N	c.92	N	c.72	N
Alpaca	Black	148	Marangani	c.629del	N	c.618	GG	c.383	N	c.354	TT	c.265	N	c.259	AA	c239-243ins	N	c.224-227del	N	c.92	N	c.72	N
Alpaca	Black	133	Marangani	c.629del	N	c.618	GG	c.383	N	c.354	TT	c.265	N	c.259	AG	c239-243ins	N	c.224-227del	Heterozygous	c.92	N	c.72	N
Alpaca	Black	151	Marangani	c.629del	N	c.618	N	c.383	N	c.354	N	c.265	N	c.259	N	c239-243ins	N	c.224-227del	N	c.92	N	c.72	N
Alpaca	Black	111	Marangani	c.629del	N	c.618	GA	c.383	N	c.354	TC	c.265	N	c.259	AG	c239-243ins	N	c.224-227del	N	c.92	N	c.72	N
Alpaca	Black	125	Marangani	c.629del	N	c.618	GA	c.383	N	c.354	TC	c.265	N	c.259	N	c239-243ins	N	c.224-227del	N	c.92	N	c.72	N
Alpaca	Black	126	Marangani	c.629del	N	c.618	GA	c.383	N	c.354	TC	c.265	N	c.259	AG	c239-243ins	N	c.224-227del	N	c.92	N	c.72	N
Alpaca	Black	190	Marangani	c.629del	N	c.618	GA	c.383	N	c.354	TC	c.265	N	c.259	AG	c239-243ins	N	c.224-227del	N	c.92	N	c.72	N
Alpaca	Black	141	Marangani	c.629del	N	c.618	N	c.383	N	c.354	TT	c.265	N	c.259	AA	c239-243ins	N	c.224-227del	N	c.92	N	c.72	N

Alpaca	Black	144	Marangani	c.629del	N	c.618	GG	c.383	N	c.354	TT	c.265	N	c.259	AA	c239-243ins	N	c.224-227del	N	c.92	N	c.72	N
Alpaca	Black	145	Marangani	c.629del	N	c.618	GA	c.383	N	c.354	TC	c.265	N	c.259	AG	c239-243ins	N	c.224-227del	N	c.92	N	c.72	N
Alpaca	Black	242	Phinaya	c.629del	N	c.618	GG	c.383	N	c.354	TT	c.265	N	c.259	AA	c239-243ins	N	c.224-227del	N	c.92	N	c.72	N
Alpaca	Black	243	Phinaya	c.629del	N	c.618	GG	c.383	N	c.354	TT	c.265	N	c.259	AA	c239-243ins	N	c.224-227del	N	c.92	N	c.72	N
Alpaca	Black	301	Phinaya	c.629del	N	c.618	GG	c.383	N	c.354	TT	c.265	N	c.259	AA	c239-243ins	N	c.224-227del	Heterozygous	c.92	N	c.72	N
Alpaca	Black	302	Phinaya	c.629del	N	c.618	GG	c.383	N	c.354	TT	c.265	N	c.259	AA	c239-243ins	N	c.224-227del	N	c.92	N	c.72	N
Alpaca	Black	303	Phinaya	c.629del	N	c.618	GG	c.383	N	c.354	TT	c.265	N	c.259	AA	c239-243ins	N	c.224-227del	N	c.92	N	c.72	N
Alpaca	Black	304	Phinaya	c.629del	N	c.618	GG	c.383	N	c.354	TT	c.265	N	c.259	AA	c239-243ins	N	c.224-227del	N	c.92	N	c.72	N
Alpaca	Black	305	Phinaya	c.629del	N	c.618	GG	c.383	N	c.354	TT	c.265	N	c.259	AA	c239-243ins	N	c.224-227del	N	c.92	N	c.72	N
Alpaca	Black	306	Phinaya	c.629del	N	c.618	GG	c.383	N	c.354	TT	c.265	N	c.259	AA	c239-243ins	N	c.224-227del	N	c.92	N	c.72	N
Alpaca	Black	307	Phinaya	c.629del	N	c.618	GA	c.383	N	c.354	TT	c.265	N	c.259	AA	c239-243ins	N	c.224-227del	N	c.92	N	c.72	N
Alpaca	Black	308	Phinaya	c.629del	N	c.618	GA	c.383	N	c.354	TC	c.265	N	c.259	AA	c239-243ins	N	c.224-227del	N	c.92	N	c.72	N
Alpaca	Black	309	Phinaya	c.629del	N	c.618	GA	c.383	N	c.354	TC	c.265	N	c.259	AA	c239-243ins	N	c.224-227del	N	c.92	N	c.72	N
Alpaca	Black	310	Phinaya	c.629del	N	c.618	GG	c.383	N	c.354	TT	c.265	N	c.259	AA	c239-243ins	N	c.224-227del	N	c.92	N	c.72	N
Alpaca	Black	311	La Raya	c.629del	N	c.618	GA	c.383	N	c.354	N	c.265	AG	c.259	AA	c239-243ins	N	c.224-227del	N	c.92	N	c.72	GC
Alpaca	Black	313	La Raya	c.629del	N	c.618	GA	c.383	N	c.354	TC	c.265	N	c.259	AA	c239-243ins	N	c.224-227del	N	c.92	N	c.72	N
Alpaca	Black	314	La Raya	c.629del	N	c.618	GA	c.383	N	c.354	TC	c.265	N	c.259	AA	c239-243ins	N	c.224-227del	N	c.92	N	c.72	N
Alpaca	Black	315	La Raya	c.629del	N	c.618	GG	c.383	N	c.354	TT	c.265	N	c.259	AA	c239-243ins	N	c.224-227del	N	c.92	N	c.72	N
Alpaca	Black	316	La Raya	c.629del	N	c.618	GA	c.383	N	c.354	TT	c.265	N	c.259	AA	c239-243ins	N	c.224-227del	N	c.92	N	c.72	N
Alpaca	Black	317	La Raya	c.629del	N	c.618	GG	c.383	N	c.354	TT	c.265	N	c.259	AA	c239-243ins	N	c.224-227del	N	c.92	N	c.72	N
Alpaca	Black	318	La Raya	c.629del	N	c.618	GG	c.383	N	c.354	TT	c.265	N	c.259	AA	c239-243ins	N	c.224-227del	Heterozygous	c.92	N	c.72	N
Alpaca	Black	319	La Raya	c.629del	N	c.618	GA	c.383	N	c.354	TC	c.265	N	c.259	AA	c239-243ins	N	c.224-227del	N	c.92	N	c.72	N
Alpaca	Black	320	La Raya	c.629del	N	c.618	GA	c.383	N	c.354	TT	c.265	N	c.259	AG	c239-243ins	N	c.224-227del	N	c.92	N	c.72	N
Alpaca	Black	321	La Raya	c.629del	N	c.618	GG	c.383	N	c.354	TT	c.265	N	c.259	AA	c239-243ins	N	c.224-227del	N	c.92	N	c.72	N
Alpaca	Black	322	La Raya	c.629del	N	c.618	GA	c.383	N	c.354	TC	c.265	N	c.259	AG	c239-243ins	N	c.224-227del	N	c.92	N	c.72	N
Alpaca	Black	323	La Raya	c.629del	N	c.618	GG	c.383	N	c.354	TT	c.265	N	c.259	AA	c239-243ins	N	c.224-227del	N	c.92	N	c.72	N
Alpaca	Black	341	La Raya	c.629del	N	c.618	GG	c.383	N	c.354	TT	c.265	N	c.259	AA	c239-243ins	N	c.224-227del	N	c.92	N	c.72	N
Alpaca	Black	342	La Raya	c.629del	N	c.618	GG	c.383	N	c.354	TT	c.265	N	c.259	AA	c239-243ins	N	c.224-227del	N	c.92	N	c.72	N
Alpaca	Black	343	La Raya	c.629del	N	c.618	GG	c.383	N	c.354	TT	c.265	N	c.259	AA	c239-243ins	N	c.224-227del	N	c.92	N	c.72	N
Alpaca	Black	344	La Raya	c.629del	N	c.618	GG	c.383	N	c.354	TT	c.265	N	c.259	AA	c239-243ins	N	c.224-227del	N	c.92	N	c.72	N
Alpaca	Black	345	La Raya	c.629del	N	c.618	GA	c.383	N	c.354	TC	c.265	N	c.259	AG	c239-243ins	N	c.224-227del	N	c.92	N	c.72	N
Alpaca	Black	346	La Raya	c.629del	N	c.618	GA	c.383	N	c.354	TC	c.265	N	c.259	AG	c239-243ins	N	c.224-227del	N	c.92	N	c.72	N
Alpaca	Black	347	La Raya	c.629del	N	c.618	GG	c.383	N	c.354	TT	c.265	N	c.259	AG	c239-243ins	N	c.224-227del	Heterozygous	c.92	N	c.72	N
Alpaca	Black	348	La Raya	c.629del	N	c.618	GA	c.383	N	c.354	TC	c.265	N	c.259	AG	c239-243ins	N	c.224-227del	N	c.92	N	c.72	N
Alpaca	Black	349	La Raya	c.629del	N	c.618	GG	c.383	N	c.354	TT	c.265	N	c.259	AA	c239-243ins	N	c.224-227del	N	c.92	N	c.72	N
Alpaca	Black	350	La Raya	c.629del	N	c.618	GG	c.383	N	c.354	TT	c.265	N	c.259	AG	c239-243ins	N	c.224-227del	N	c.92	CT	c.72	N
Alpaca	Black	351	La Raya	c.629del	N	c.618	GG	c.383	N	c.354	TT	c.265	N	c.259	AA	c239-243ins	N	c.224-227del	N	c.92	N	c.72	N
Alpaca	Black	352	La Raya	c.629del	N	c.618	GA	c.383	N	c.354	TC	c.265	N	c.259	AG	c239-243ins	N	c.224-227del	N	c.92	N	c.72	N
Alpaca	Black	353	Nuñoa	c.629del	N	c.618	GA	c.383	N	c.354	TC	c.265	N	c.259	AG	c239-243ins	N	c.224-227del	N	c.92	N	c.72	N
Alpaca	Black	355	Nuñoa	c.629del	N	c.618	GG	c.383	TC	c.354	TT	c.265	N	c.259	AG	c239-243ins	N	c.224-227del	N	c.92	N	c.72	N
Alpaca	Black	356	Nuñoa	c.629del	N	c.618	GG	c.383	N	c.354	TT	c.265	N	c.259	AG	c239-243ins	N	c.224-227del	N	c.92	N	c.72	N
Alpaca	Black	357	Nuñoa	c.629del	N	c.618	GG	c.383	N	c.354	TT	c.265	N	c.259	AA	c239-243ins	N	c.224-227del	N	c.92	N	c.72	N
Alpaca	Black	358	Nuñoa	c.629del	N	c.618	GG	c.383	N	c.354	TT	c.265	N	c.259	AA	c239-243ins	N	c.224-227del	N	c.92	N	c.72	N
Alpaca	Black	359	Nuñoa	c.629del	N	c.618	GG	c.383	TC	c.354	TT	c.265	N	c.259	AG	c239-243ins	N	c.224-227del	N	c.92	N	c.72	N
Alpaca	Black	360	Nuñoa	c.629del	N	c.618	GG	c.383	TC	c.354	TT	c.265	N	c.259	AG	c239-243ins	N	c.224-227del	N	c.92	N	c.72	N
Alpaca	Black	361	Nuñoa	c.629del	N	c.618	GG	c.383	N	c.354	TT	c.265	N	c.259	AA	c239-243ins	N	c.224-227del	N	c.92	N	c.72	N
Alpaca	Black	362	Nuñoa	c.629del	N	c.618	GG	c.383	N	c.354	TT	c.265	N	c.259	AA	c239-243ins	N	c.224-227del	N	c.92	N	c.72	N

Alpaca	Black	363	Nuñoa	c.629del	N	c.618	GG	c.383	TC	c.354	TT	c.265	N	c.259	AG	c239-243ins	N	c.224-227del	N	c.92	N	c.72	N
Alpaca	Black	364	Nuñoa	c.629del	N	c.618	GG	c.383	N	c.354	TT	c.265	N	c.259	AA	c239-243ins	N	c.224-227del	N	c.92	N	c.72	N
Alpaca	Black	365	Nuñoa	c.629del	N	c.618	GA	c.383	N	c.354	TT	c.265	N	c.259	AG	c239-243ins	N	c.224-227del	N	c.92	N	c.72	N
Alpaca	Black	366	Nuñoa	c.629del	N	c.618	GG	c.383	N	c.354	TT	c.265	N	c.259	AA	c239-243ins	N	c.224-227del	N	c.92	N	c.72	N
Alpaca	Black	367	Nuñoa	c.629del	N	c.618	N	c.383	N	c.354	N	c.265	N	c.259	N	c239-243ins	N	c.224-227del	N	c.92	N	c.72	N
Alpaca	Black	368	Nuñoa	c.629del	N	c.618	GG	c.383	N	c.354	TT	c.265	N	c.259	AG	c239-243ins	N	c.224-227del	Heterozygous	c.92	N	c.72	N
Alpaca	Black	369	Nuñoa	c.629del	N	c.618	GA	c.383	N	c.354	TT	c.265	N	c.259	AG	c239-243ins	N	c.224-227del	N	c.92	N	c.72	N
Alpaca	Black	370	Nuñoa	c.629del	N	c.618	GG	c.383	N	c.354	TT	c.265	N	c.259	AA	c239-243ins	N	c.224-227del	N	c.92	N	c.72	N
Alpaca	Black	371	Nuñoa	c.629del	Absent	c.618	AA	c.383	CC	c.354	CC	c.265	GG	c.259	GG	c239-243ins	Absent	c.224-227del	Absent	c.92	TT	c.72	CC
Alpaca	Black	372	Nuñoa	c.629del	N	c.618	GA	c.383	CC	c.354	TC	c.265	N	c.259	AG	c239-243ins	N	c.224-227del	Absent	c.92	N	c.72	N
Alpaca	Black	373	Nuñoa	c.629del	N	c.618	GA	c.383	CC	c.354	TT	c.265	N	c.259	AG	c239-243ins	N	c.224-227del	Absent	c.92	N	c.72	N
Alpaca	Black	374	Nuñoa	c.629del	N	c.618	N	c.383	CC	c.354	TC	c.265	N	c.259	GG	c239-243ins	N	c.224-227del	Absent	c.92	N	c.72	N
Alpaca	Black	375	Nuñoa	c.629del	Absent	c.618	GA	c.383	CC	c.354	CC	c.265	AG	c.259	GG	c239-243ins	Absent	c.224-227del	Absent	c.92	TT	c.72	GC
Alpaca	Black	376	Nuñoa	c.629del	Absent	c.618	AA	c.383	CC	c.354	CC	c.265	GG	c.259	GG	c239-243ins	Absent	c.224-227del	Absent	c.92	TT	c.72	CC
Alpaca	Black	377	Nuñoa	c.629del	Absent	c.618	GG	c.383	CC	c.354	TT	c.265	GG	c.259	AA	c239-243ins	Absent	c.224-227del	Absent	c.92	TT	c.72	CC
Alpaca	Black	378	Nuñoa	c.629del	Absent	c.618	GG	c.383	CC	c.354	TT	c.265	GG	c.259	AA	c239-243ins	Absent	c.224-227del	Absent	c.92	N	c.72	N
Alpaca	Black	379	Nuñoa	c.629del	Absent	c.618	GG	c.383	TC	c.354	TT	c.265	GG	c.259	AG	c239-243ins	Absent	c.224-227del	Absent	c.92	TT	c.72	CC
Alpaca	Black	380	Nuñoa	c.629del	Absent	c.618	GG	c.383	CC	c.354	TT	c.265	GG	c.259	AA	c239-243ins	Absent	c.224-227del	Absent	c.92	TT	c.72	CC
Alpaca	Black	381	Nuñoa	c.629del	Absent	c.618	GA	c.383	CC	c.354	TC	c.265	GG	c.259	GG	c239-243ins	Absent	c.224-227del	Heterozygous	c.92	TT	c.72	CC
Alpaca	Black	382	Nuñoa	c.629del	Absent	c.618	GA	c.383	CC	c.354	TC	c.265	GG	c.259	AG	c239-243ins	Absent	c.224-227del	Absent	c.92	TT	c.72	CC
Alpaca	Black	383	Nuñoa	c.629del	N	c.618	N	c.383	CC	c.354	N	c.265	N	c.259	N	c239-243ins	Heterozygous	c.224-227del	N	c.92	TT	c.72	CC
Alpaca	Black	384	Nuñoa	c.629del	Absent	c.618	GA	c.383	CC	c.354	TC	c.265	GG	c.259	AG	c239-243ins	Absent	c.224-227del	Absent	c.92	TT	c.72	CC
Alpaca	Black	385	Nuñoa	c.629del	N	c.618	N	c.383	CC	c.354	N	c.265	N	c.259	N	c239-243ins	N	c.224-227del	N	c.92	TT	c.72	CC
Alpaca	Black	386	Nuñoa	c.629del	Absent	c.618	GA	c.383	CC	c.354	TC	c.265	N	c.259	AG	c239-243ins	Absent	c.224-227del	Absent	c.92	N	c.72	N
Alpaca	Black	387	Nuñoa	c.629del	Absent	c.618	GG	c.383	CC	c.354	TT	c.265	GG	c.259	AA	c239-243ins	Absent	c.224-227del	Absent	c.92	N	c.72	N
Alpaca	Black	388	Nuñoa	c.629del	N	c.618	N	c.383	CC	c.354	N	c.265	N	c.259	N	c239-243ins	N	c.224-227del	N	c.92	N	c.72	N
Alpaca	Black	389	Nuñoa	c.629del	Absent	c.618	GA	c.383	CC	c.354	TC	c.265	N	c.259	AG	c239-243ins	N	c.224-227del	N	c.92	TT	c.72	N
Alpaca	Black	390	Nuñoa	c.629del	Absent	c.618	GA	c.383	CC	c.354	TC	c.265	GG	c.259	AG	c239-243ins	Absent	c.224-227del	Absent	c.92	TT	c.72	CC
Alpaca	Black	391	Nuñoa	c.629del	Absent	c.618	GG	c.383	CC	c.354	TT	c.265	GG	c.259	AA	c239-243ins	Absent	c.224-227del	Absent	c.92	TT	c.72	CC
Alpaca	Black	392	Nuñoa	c.629del	Absent	c.618	GG	c.383	CC	c.354	TT	c.265	GG	c.259	AA	c239-243ins	Absent	c.224-227del	Absent	c.92	N	c.72	N
Guanaco	Brown	1	Nuñoa	c.629del	N	c.618	GG	c.383	N	c.354	TT	c.265	N	c.259	N	c239-243ins	N	c.224-227del	N	c.92	N	c.72	N
Guanaco	Brown	2	Nuñoa	c.629del	N	c.618	GG	c.383	N	c.354	TT	c.265	N	c.259	N	c239-243ins	N	c.224-227del	N	c.92	N	c.72	N
Guanaco	Brown	3	Nuñoa	c.629del	N	c.618	GG	c.383	TC	c.354	TT	c.265	N	c.259	N	c239-243ins	N	c.224-227del	N	c.92	N	c.72	N
Llama	Brown	1	Nuñoa	c.629del	N	c.618	GG	c.383	N	c.354	TT	c.265	N	c.259	N	c239-243ins	N	c.224-227del	Heterozygous	c.92	N	c.72	N
Llama	Brown	2	Nuñoa	c.629del	N	c.618	GG	c.383	N	c.354	TT	c.265	N	c.259	AG	c239-243ins	N	c.224-227del	N	c.92	N	c.72	N
Llama	Brown	3	Nuñoa	c.629del	N	c.618	GG	c.383	TC	c.354	TT	c.265	N	c.259	N	c239-243ins	N	c.224-227del	N	c.92	N	c.72	N
Llama	Brown	4	Nuñoa	c.629del	N	c.618	GG	c.383	N	c.354	TT	c.265	N	c.259	AG	c239-243ins	N	c.224-227del	N	c.92	N	c.72	N
Llama	Brown	5	Nuñoa	c.629del	N	c.618	GG	c.383	N	c.354	TT	c.265	N	c.259	N	c239-243ins	N	c.224-227del	Heterozygous	c.92	N	c.72	N
Vicuña	Brown	1	Marangani	c.629del	N	c.618	N	c.383	N	c.354	N	c.265	N	c.259	N	c239-243ins	N	c.224-227del	N	c.92	N	c.72	N
Vicuña	Brown	2	Marangani	c.629del	N	c.618	N	c.383	N	c.354	TC	c.265	N	c.259	N	c239-243ins	N	c.224-227del	N	c.92	N	c.72	N
Vicuña	Brown	3	Marangani	c.629del	N	c.618	N	c.383	N	c.354	N	c.265	N	c.259	N	c239-243ins	N	c.224-227del	N	c.92	N	c.72	N
Vicuña	Brown	4	Marangani	c.629del	N	c.618	N	c.383	N	c.354	N	c.265	N	c.259	N	c239-243ins	N	c.224-227del	N	c.92	N	c.72	N
Vicuña	Brown	5	Marangani	c.629del	N	c.618	N	c.383	N	c.354	N	c.265	N	c.259	N	c239-243ins	N	c.224-227del	N	c.92	N	c.72	N
Vicuña	Brown	6	Marangani	c.629del	N	c.618	N	c.383	N	c.354	TC	c.265	N	c.259	N	c239-243ins	N	c.224-227del	N	c.92	N	c.72	N
Vicuña	Brown	7	Marangani	c.629del	N	c.618	N	c.383	N	c.354	N	c.265	N	c.259	N	c239-243ins	N	c.224-227del	N	c.92	N	c.72	N
Vicuña	Brown	8	Marangani	c.629del	N	c.618	N	c.383	N	c.354	N	c.265	N	c.259	N	c239-243ins	N	c.224-227del	N	c.92	N	c.72	N
Vicuña	Brown	9	Marangani	c.629del	N	c.618	GA	c.383	N	c.354	N	c.265	AG	c.259	N	c239-243ins	N	c.224-227del	N	c.92	N	c.72	GC
