# Supplementary material for: MC1R Gene Variants and Their Relationship with Coat Color in South American Camelids
Source: ScientificWorldJournal. 2023 Aug 30;2023:4871135. doi: 10.1155/2023/4871135 (PMC10541998; doi:10.1155/2023/4871135)
Supplement: Supplementary Materials — Figure S1: some coat color in alpacas and llamas. Figure S2: structural organization of the MC1R gene with identified polymorphisms. Table S1: information from the animals used in this study, with significant polymorphisms in statistical analysis for the trait analyzed. Table S2: information from animals used in this study, with polymorphisms found in the MC1R gene that were not significant in the statistical analysis. Table S3: distribution of genotypes for polymorphisms among different color phenotypes. Table S4: statistical analyses used in the population studied. Table S5: frequency of haplotypes in the population studied. [file 4871135.f1.zip › Table S1.pdf]

Table 1S. Distribution of genotypes for polymorphisms among the different color phenotypes.

SNP observed	Allele	Alpacas phenotype				Brown llama	Brown guanaco	Brown vicuña
		White	Brown	LF	Black			
**c.72G>C**	GG	0	0	0	0	0	0	0
	GC	1	5	0	3	0	0	1
	CC	108	37	9	12	0	0	0
**c.82A>G**	AA	10	18	0	43	5	3	0
	AG	12	22	2	34	0	0	0
	GG	92	5	7	5	0	0	0
**c.92C>T**	CC	0	0	0	0	0	0	0
	CT	0	0	0	1	0	0	0
	TT	115	41	9	14	0	0	0
**c.126T>C**	TT	10	20	2	43	5	3	0
	TC	12	23	0	35	0	0	0
	CC	106	5	7	5	0	0	0
**c.224-227del**	Del	0	0	2	0	0	0	0
	Het	22	0	5	7	2	0	0
	No del	90	42	8	17	0	0	0
**c.239-243ins**	Ins	10	5	0	0	0	0	0
	Het	5	8	0	1	0	0	0
	No ins	91	27	12	15	0	0	0
**c.243C>T**	CC	0	0	0	0	0	0	0
	CT	1	0	0	3	0	0	0
	TT	93	40	12	14	0	0	0
**c.259A>G**	AA	7	15	0	49	0	0	0
	AG	5	19	3	33	2	0	0
	GG	92	8	11	5	0	0	0
**c.265A>G**	AA	0	0	0	0	0	0	0
	AG	1	3	0	3	0	0	1
	GG	92	92	12	13	0	0	0
**c.354T>C**	TT	11	16	2	57	5	3	0
	TC	39	21	8	26	0	0	2
	CC	65	5	5	3	0	0	0
**c.376G>A**	GG	6	14	0	34	0	0	0
	GA	9	30	3	49	2	0	0

	AA	129	11	11	5	0	0	0
**c.383T>C**	TT	0	0	0	0	0	0	0
	TC	10	7	0	6	1	1	0
	CC	129	39	14	21	0	0	0
**c.618G>A**	GG	11	14	2	49	5	3	0
	GA	32	15	7	35	0	0	1
	AA	63	5	5	2	0	0	0
**c.629Tdel**	Del	2	10	0	0	0	0	0
	Het	0	6	0	0	0	0	0
	No del	91	23	12	16	0	0	0
**c.901C>T**	CC	11	26	2	58	0	0	0
	CT	54	19	6	26	5	3	9
	TT	82	5	5	2	0	0	0
**c.933G>A**	GG	10	17	2	44	5	3	0
	GA	39	25	6	35	0	0	0
	AA	92	5	5	4	0	0	0
**5’c. -42C>G**	CC	10	19	0	43	5	3	0
	CG	13	23	2	34	0	0	0
	GG	101	5	7	5	0	0	0
**3’UTR +8T>C**	TT	10	17	2	43	5	3	0
	TC	39	24	6	36	0	0	0
	CC	86	5	4	4	0	0	0
**3’UTR +170 G>C**	GG	9	19	2	44	5	3	0
	GC	39	25	6	40	0	0	1
	CC	8	5	4	3	0	0	0

LF: Light fawn color. Het: heterozygous.
