# Supplementary material for: MC1R Gene Variants and Their Relationship with Coat Color in South American Camelids
Source: ScientificWorldJournal. 2023 Aug 30;2023:4871135. doi: 10.1155/2023/4871135 (PMC10541998; doi:10.1155/2023/4871135)
Supplement: Supplementary Materials — Figure S1: some coat color in alpacas and llamas. Figure S2: structural organization of the MC1R gene with identified polymorphisms. Table S1: information from the animals used in this study, with significant polymorphisms in statistical analysis for the trait analyzed. Table S2: information from animals used in this study, with polymorphisms found in the MC1R gene that were not significant in the statistical analysis. Table S3: distribution of genotypes for polymorphisms among different color phenotypes. Table S4: statistical analyses used in the population studied. Table S5: frequency of haplotypes in the population studied. [file 4871135.f1.zip › Table_S4. Statistical analysis.pdf]

Table S4. Statistical analyses evaluated in the alpaca population.

CHR	SNP	BP	A1	F_A	F_U	A2	CHISQ	P	OR	Cutoff	Significant
NW_021969788.1	.	137549	T	0.5645	0.1277	C	59.22	1.411E-14	8.858	2.7533E-05	TRUE
NW_021969788.1	.	137330	A	0.6042	0.1691	G	43.34	4.591E-11	7.502	2.7563E-05	TRUE
NW_021969788.1	.	138324	G	0.6471	0.1715	A	39.34	3.553E-10	8.855	2.7579E-05	TRUE
NW_021969788.1	.	137517	T	0.587	0.1691	C	39.11	4.003E-10	6.984	2.7594E-05	TRUE
NW_021969788.1	.	137491	G	0.587	0.1691	A	39.11	4.003E-10	6.984	2.7609E-05	TRUE
NW_021969788.1	.	138491	C	0.625	0.1715	G	34.45	4.364E-09	8.05	2.7655E-05	TRUE
NW_021969788.1	.	138368	C	0.625	0.1715	T	34.45	4.364E-09	8.05	2.767E-05	TRUE
NW_021969788.1	.	138074	C	0.7333	0.2409	T	31.88	1.638E-08	8.667	2.7685E-05	TRUE

CHR: Chromosome ID. BP: Base pair. A1: Reference nucleotide. A2: Change in nucleotide. F_A: Allele frequency in cases. F_U: Allele frequency in controls. CHISQ: Chi square. P: P-value. OR: Odds ratio.
