# Supplementary material for: MC1R Gene Variants and Their Relationship with Coat Color in South American Camelids
Source: ScientificWorldJournal. 2023 Aug 30;2023:4871135. doi: 10.1155/2023/4871135 (PMC10541998; doi:10.1155/2023/4871135)
Supplement: Supplementary Materials — Figure S1: some coat color in alpacas and llamas. Figure S2: structural organization of the MC1R gene with identified polymorphisms. Table S1: information from the animals used in this study, with significant polymorphisms in statistical analysis for the trait analyzed. Table S2: information from animals used in this study, with polymorphisms found in the MC1R gene that were not significant in the statistical analysis. Table S3: distribution of genotypes for polymorphisms among different color phenotypes. Table S4: statistical analyses used in the population studied. Table S5: frequency of haplotypes in the population studied. [file 4871135.f1.zip › Table_S5hap.docx]

**Table S5:** Haplotype frequency in analyzed alpacas (N=166), llamas (N=5), guanacos (N=83) and vicuñas (N=7), Only polymorphisms significant to the analysis are listed in the table, but the analysis was performed taking into account all polymorphisms (19).

| Haplotypes  (Number of animals) | % | c.82  A>G | c.126  T>C | c.376  G>A | c.901  C>T | c.933  G>A | 5’c.  -42  C>G | 3’UTR  +5  T>C | 3’UTR  +170 G>C | Specie |
| --- | --- | --- | --- | --- | --- | --- | --- | --- | --- | --- |
| H1 (132) | 37.61 | A | T | A | C | G | C | T | G | WA, BlA, BrA, LFA, Ll, G, V |
| H2 (94) | 26.78 | G | C | G | T | A | G | C | C | WA, BlA, BrA, LFA |
| H3 (24) | 6.84 | N | N | G | N | N | G | T | C | WA, BlA, LFA |
| H4 (14) | 3.99 | A | T | N | C | G | C | T | G | WA, BlA, BrA, Ll, G |
| H5 (11) | 3.13 | G | C | G | T | A | G | C | G | WA |
| H6 (7) | 1.99 | N | N | N | T | N | C | T | G | WA, BlA, BrA |
| H7 (6) | 1.71 | A | T | A | C | G | C | T | G | BrA |
| H8 (6) | 1.71 | A | T | G | C | G | G | C | C | WA |
| H9 (5) | 1.42 | G | C | G | C | A | G | C | G | WA |
| H10 (4) | 1.14 | N | N | N | C | N | G | C | C | WA, BlA, V |
| H11 (4) | 1.14 | G | C | G | C | A | G | C | C | WA, BlA |
| H12 (4) | 1.14 | G | C | G | C | G | G | C | C | WA |
| H13 (3) | 0.85 | G | T | A | C | G | C | T | G | BrA |
| H14 (2) | 0.57 | A | T | A | C | G | C | T | G | BrA |
| H15 (2) | 0.57 | A | T | A | C | G | C | T | G | BlA, BrA |
| H16 (2) | 0.57 | A | T | A | C | G | C | T | G | BrA |
| H17 (2) | 0.57 | N | T | A | C | G | C | T | G | WA |
| H18 (2) | 0.57 | G | C | G | C | G | G | C | C | WA |
| H19 (2) | 0.57 | N | N | N | N | N | C | T | G | BlA |
| H20 (2) | 0.57 | G | C | G | C | G | G | T | G | WA |
| H21 (2) | 0.57 | G | T | G | T | A | G | C | C | WA |
| H22 (2) | 0.57 | A | T | N | C | G | C | T | G | Ll |
| H23 (1) | 0.28 | G | C | C | T | A | G | C | G | WA |
| H24 (1) | 0.28 | G | C | G | C | A | N | N | G | WA |
| H25 (1) | 0.28 | A | T | A | C | G | C | T | G | BrA |
| H26 (1) | 0.28 | N | T | A | C | G | C | T | G | BrA |
| H27 (1) | 0.28 | A | T | G | C | G | C | T | G | BrA |
| H28 (1) | 0.28 | A | T | G | C | G | C | T | G | BrA |
| H29 (1) | 0.28 | A | T | A | C | G | N | N | N | BlA |
| H30 (1) | 0.28 | A | T | A | T | G | G | C | C | WA |
| H31 (1) | 0.28 | G | N | G | C | G | G | T | G | BrA |
| H32 (1) | 0.28 | G | C | G | C | G | N | T | N | WA |
| H33 (1) | 0.28 | C | C | G | C | A | C | T | G | BlA |
| H34 (1) | 0.28 | N | N | A | N | A | C | T | G | BlA |
| H35 (1) | 0.28 | N | N | N | T | N | N | N | N | WA |
| H36 (1) | 0.28 | N | N | N | T | A | G | C | C | WA |
| H37 (1) | 0.28 | G | T | G | C | G | C | T | G | WA |
| H38 (1) | 0.28 | A | T | A | C | G | G | C | C | WA |
| H39 (1) | 0.28 | A | T | G | C | G | C | T | G | WA |
| H40 (1) | 0.28 | G | C | G | T | A | G | C | C | WA |
| H41 (1) | 0.28 | A | T | A | C | G | C | T | G | BrA |

WA= White alpaca, BrA= Brown alpaca, BlA= Black alpaca, LFA= LF alpaca, G= Guanaco, Ll= Llama, V= Vicuña
