# Supplementary figures and images for: MC1R Gene Variants and Their Relationship with Coat Color in South American Camelids
Source: ScientificWorldJournal. 2023 Aug 30;2023:4871135. doi: 10.1155/2023/4871135 (PMC10541998; doi:10.1155/2023/4871135)

Supplementary Figure 2: Structural organization of MC1R gene.

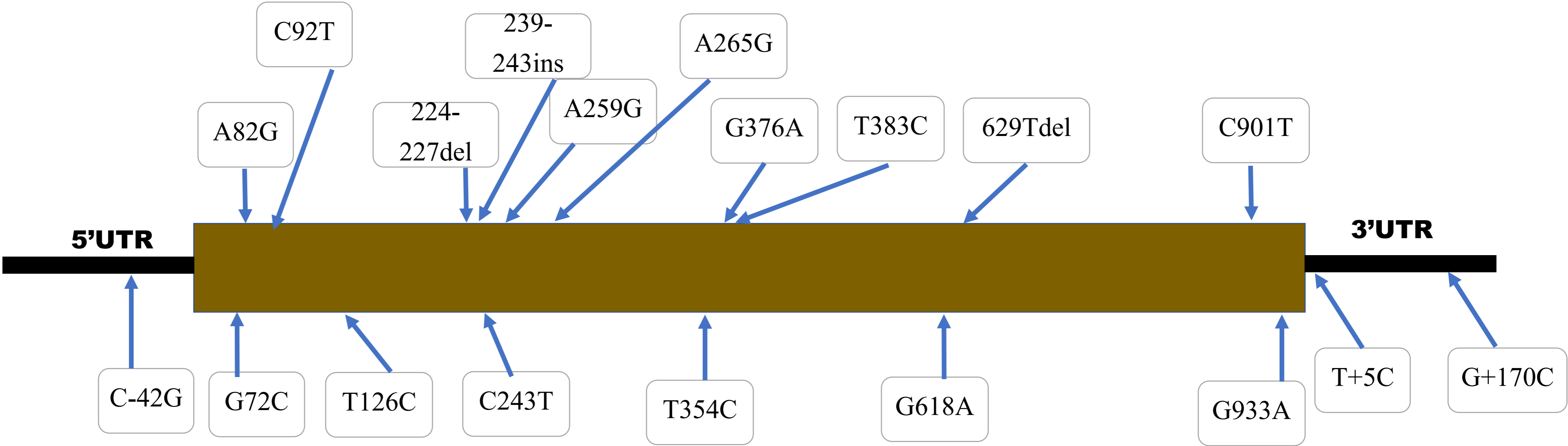

Supplement: Supplementary Materials — Figure S1: some coat color in alpacas and llamas. Figure S2: structural organization of the MC1R gene with identified polymorphisms. Table S1: information from the animals used in this study, with significant polymorphisms in statistical analysis for the trait analyzed. Table S2: information from animals used in this study, with polymorphisms found in the MC1R gene that were not significant in the statistical analysis. Table S3: distribution of genotypes for polymorphisms among different color phenotypes. Table S4: statistical analyses used in the population studied. Table S5: frequency of haplotypes in the population studied. [file 4871135.f1.zip › Fig S2. MC1R gene.pdf]

**Figure S1:** Coat color in alpacas and llamas

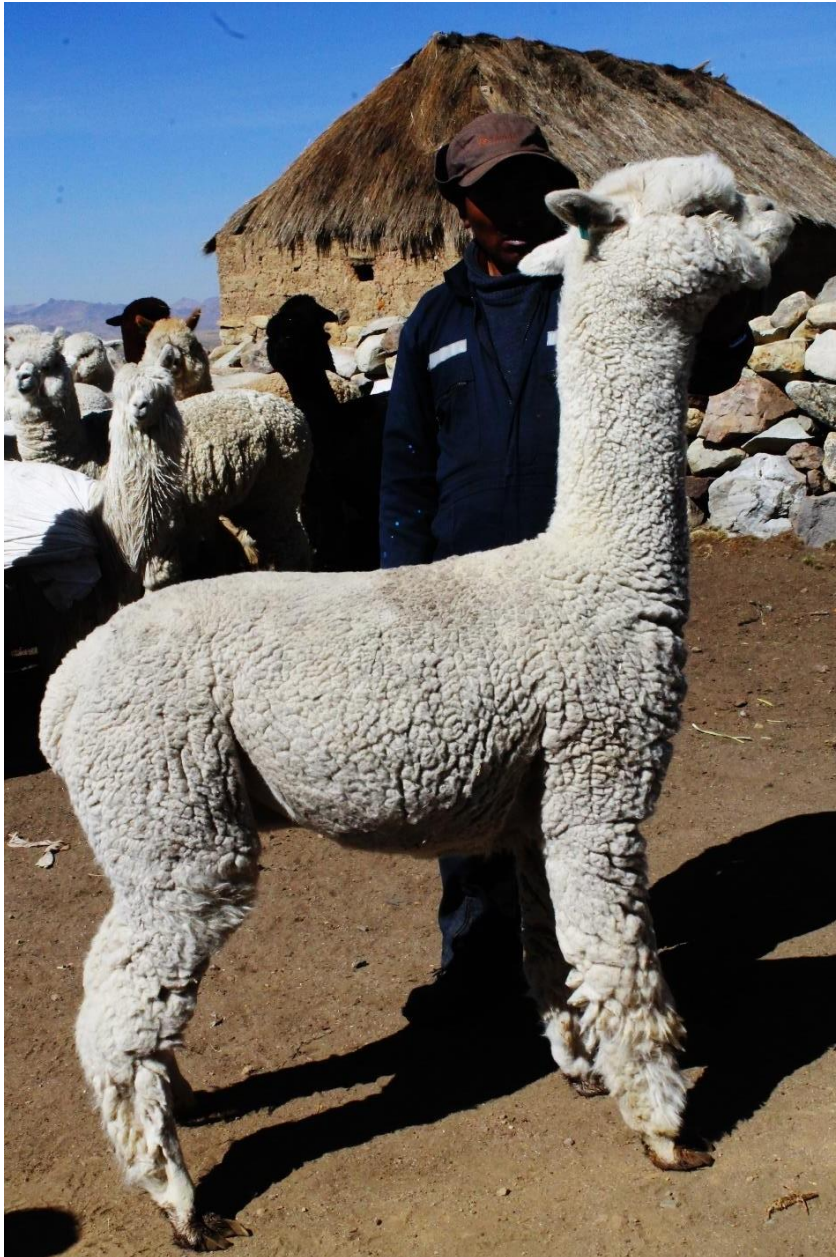

White alpaca

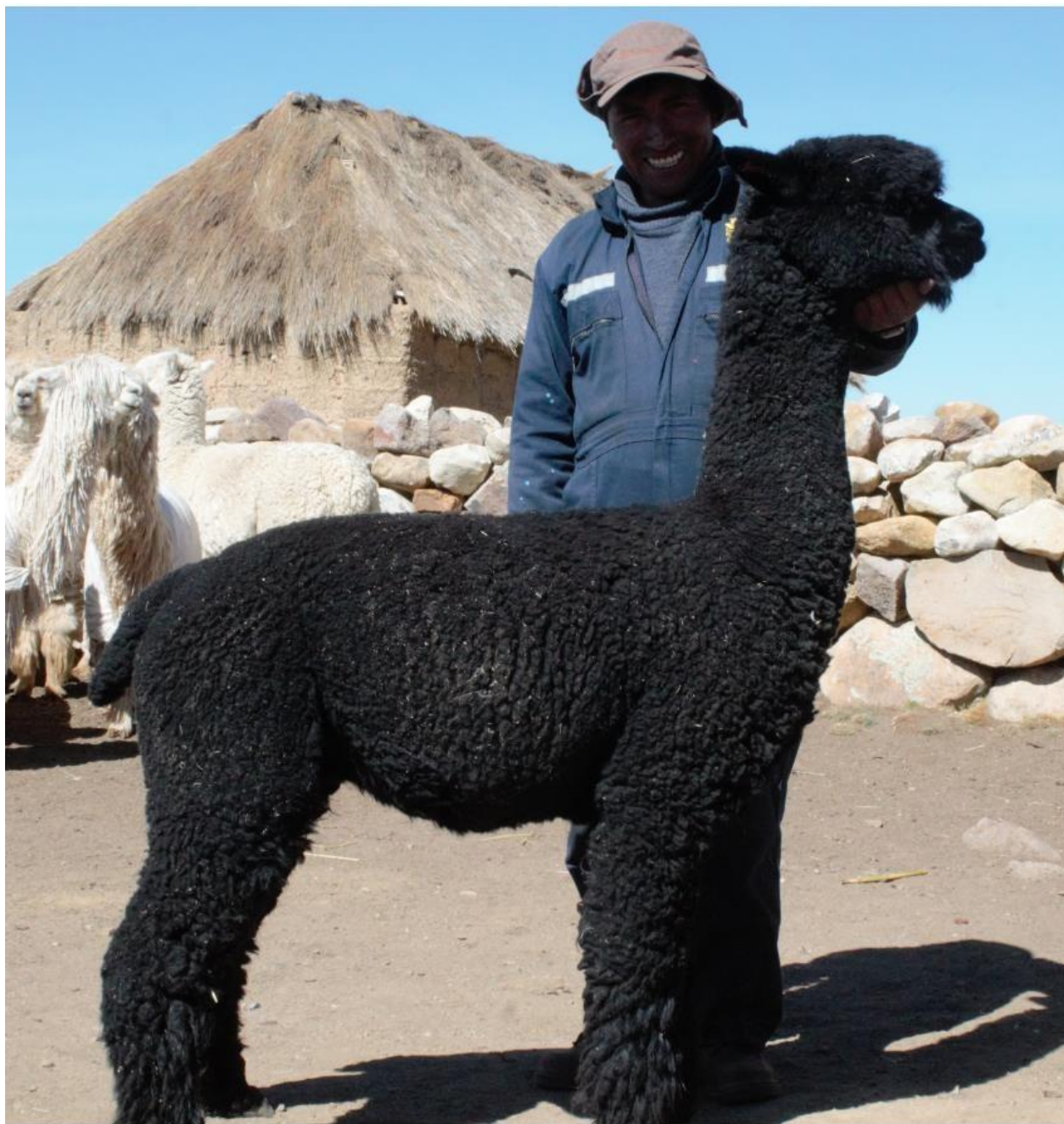

Black alpaca

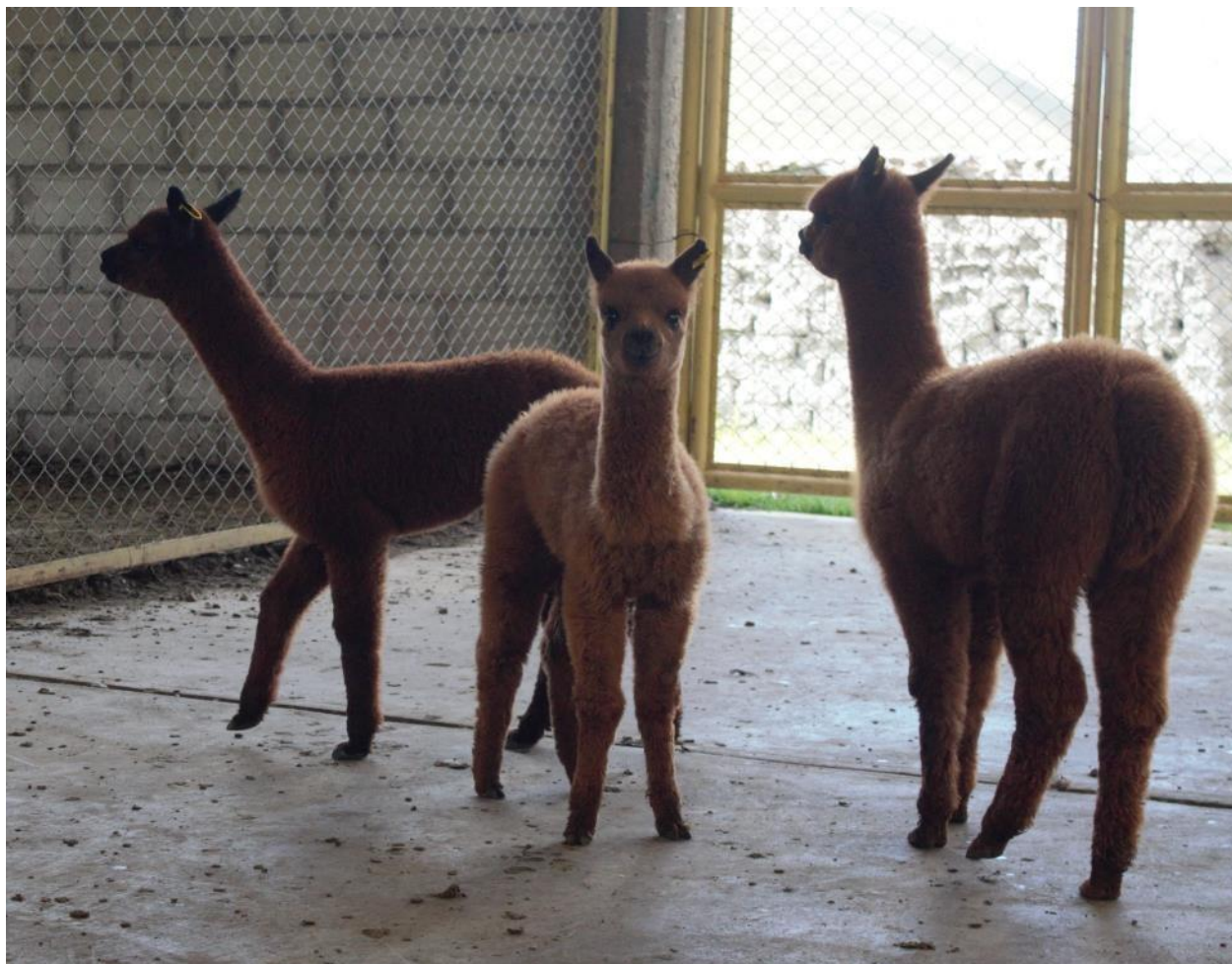

Brown alpacas

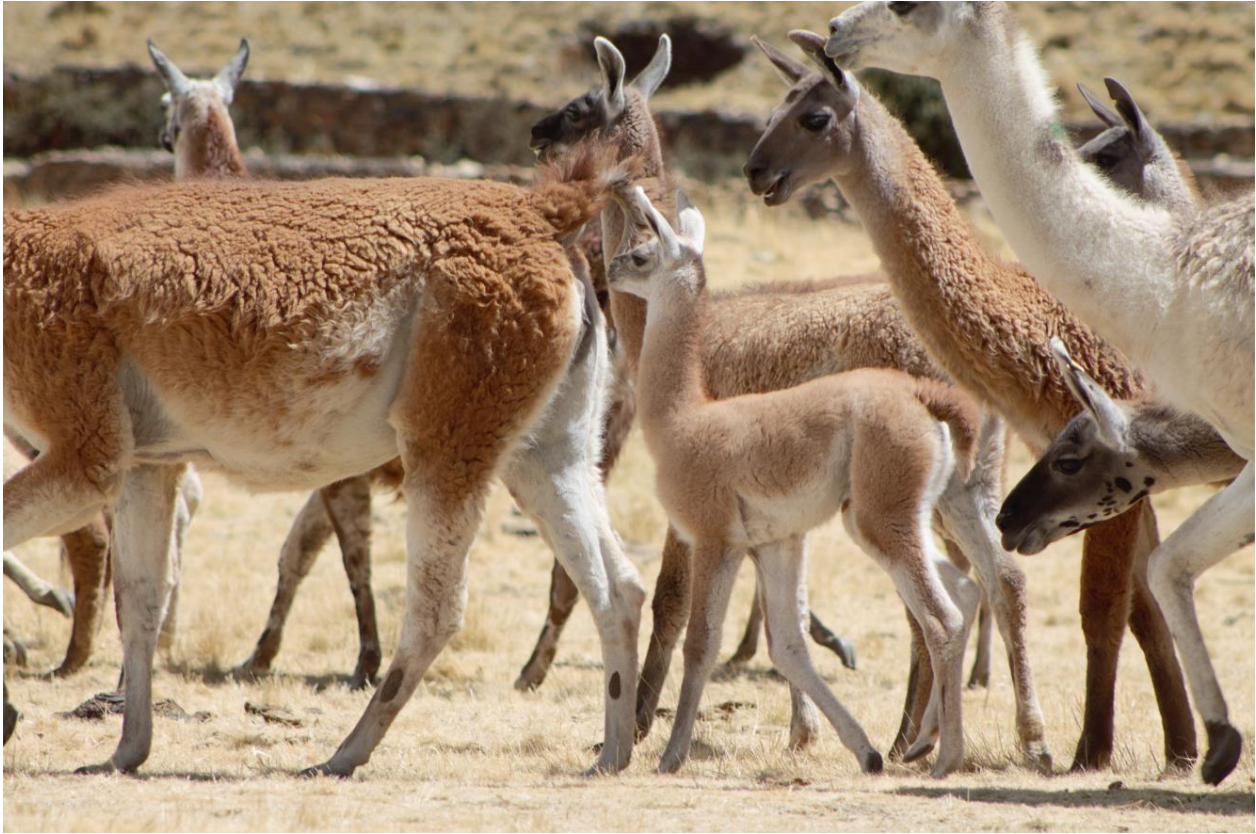

K'ara llamas

Supplement: Supplementary Materials — Figure S1: some coat color in alpacas and llamas. Figure S2: structural organization of the MC1R gene with identified polymorphisms. Table S1: information from the animals used in this study, with significant polymorphisms in statistical analysis for the trait analyzed. Table S2: information from animals used in this study, with polymorphisms found in the MC1R gene that were not significant in the statistical analysis. Table S3: distribution of genotypes for polymorphisms among different color phenotypes. Table S4: statistical analyses used in the population studied. Table S5: frequency of haplotypes in the population studied. [file 4871135.f1.zip › Figure S1 Coat color in camelids.pdf]
